# Phosphoethanolamine modification of lipid A found in five distinct *Akkermansia* species

**DOI:** 10.1128/spectrum.00682-26

**Published:** 2026-06-22

**Authors:** Silas Porter Crenna, Arianna Lagoudakis, Angela M. Jackson, Kenneth Weke, Georgia Tiegs, David R. Goodlett, Lauren E. Davey, Helena Pětrošová

**Affiliations:** 1 University of Victoria Genome BC Proteomics Centre 271484 , Victoria, BC, Canada; 2 Department of Biochemistry and Microbiology, University of Victoria 175101, Victoria, BC, Canada; Institute of Parasitology, Biology Centre, ASCR, Ceske Budejovice, Czechia

**Keywords:** *Akkermansia*, lipid A, phosphoethanolamine, lipid A modifications, gut microbiota, mass spectrometry, colistin, lipidomics, proteomics, structure function, MALDI

## Abstract

*Akkermansia* is an important, diderm gut microbe associated with positive health outcomes. Recent advances in *Akkermansia* genomics have revealed great diversity in the genus, leading to the discovery of novel species and subspecies. Our knowledge of their individual interactions with the host is limited. One of the key questions concerning diderm commensals centers on their lipopolysaccharide and its outer membrane component, lipid A. Commensals must employ strategies that prevent eliciting lipid A-induced inflammation and resist cationic antimicrobial peptides, allowing them to colonize the host. In this study, lipid A was examined across representative isolates of five *Akkermansia* species to lay the foundation for investigating these host-microbe interactions across the genus. Lipid A of *A. muciniphila, A. massiliensis*, *A. biwaensis*, *A. ignis*, and *A. durhamii* (*N* = 55) were analyzed by Matrix-Assisted Laser Desorption/Ionization mass spectrometry. All *Akkermansia* species produced lipid A modified with phosphoethanolamine (pEtN), which is associated with antimicrobial resistance and immune evasion. The relative abundance of modified lipid A varied among the species and positively correlated with decreased susceptibility to the antimicrobial peptide colistin. Intriguingly, the relative abundance of modified lipid A was independent of colistin presence. Instead, it increased as bacteria entered the stationary growth phase. Using proteomics approaches, cell envelope biogenesis-associated proteins were identified that could be involved in the associated outer membrane remodeling. To summarize, this study reports a novel lipid A modification and associated phenotypes in *Akkermansia*. Further work is warranted to determine its significance in the *Akkermansia* interactions with host cells in the gut.

**IMPORTANCE:**

*Akkermansia* is a beneficial gut bacterium linked to metabolic and immune health. Yet we still know little about how different *Akkermansia* species interact with the human host. One key factor in these interactions is lipopolysaccharide (LPS), a molecule on the bacterial surface that can either stimulate or dampen inflammation. Here, we show that all investigated species share a chemical modification of LPS not previously reported in *Akkermansia*. This modification is associated with decreased sensitivity to antimicrobial peptide colistin and becomes more abundant as bacteria enter stationary phase, suggesting it is part of a regulated adaptation strategy rather than simply a response to antibiotic exposure. By identifying and functionally examining the modification, this work provides new insight into mechanisms that may support persistence in the intestinal environment.

## INTRODUCTION

*Akkermansia* is a prevalent member of the human gut microbiota, associated with protection against metabolic disease, neurological conditions, and enhanced response to cancer treatment (1–5). How *Akkermansia* interacts with the host is not fully understood; however, a number of metabolites and cell components that contribute to its beneficial effects have been identified (5–8). Efforts to isolate and sequence *Akkermansia* strains have revealed significant diversity within the genus, and recent changes to *Akkermansia* taxonomy have led to the recognition of multiple distinct species and subspecies (9–11). Certain species have been found to correlate with various health outcomes, but their distinct characteristics are just beginning to be understood (11, 12).

A key question concerning diderm commensal bacteria is how they colonize their hosts without inducing extensive inflammation through their surface lipopolysaccharide (LPS). This microbe-associated molecular pattern is localized in the bacterial outer membrane and consists of three parts: lipid A, core antigen, and O-antigen. The canonical lipid A from *Escherichia coli* is a potent activator of the Toll-like receptor 4/Myeloid Differentiation Protein 2 complex (TLR4/MD2), eliciting strong activation of innate immune cascades (13). Work examining how commensals avoid these responses has revealed a range of mechanisms, including alterations to the number of lipid A acyl chains or removal of phosphate groups (14, 15). The altered lipid A can, in turn, inhibit innate immune signaling, acting as an antagonist of TLR4/MD2 (16). The highly charged phosphate backbone of lipid A can also undergo the addition of groups to repress host detection (17). These additional modifications to phosphate groups of lipid A can also increase resistance to host-produced cationic antimicrobial peptides (18). For example, the addition of the positively charged moiety phosphoethanolamine (pEtN) decreases the binding of cationic antimicrobial peptides to lipid A phosphates, protecting the bacterial membrane (19). In many pathogens, this modification is inducible and regulated by two-component systems in response to environmental stress (20, 21). However, the regulatory mechanisms governing lipid A remodeling in gut commensals remain less clear. Gut commensals also often lack the outermost portion of LPS, the O-antigen, and instead produce shorter and less immunogenic lipooligosaccharide (LOS) (22, 23). These modifications can tune the interaction with host receptors and modulate the downstream responses, supporting their existence in the gut. Studies also suggest that maintaining a balance between agonistic and antagonistic LPS among gut commensals contributes to intestinal health. While a high abundance of bacterial species with endotoxic LPS can lead to or exacerbate intestinal inflammation, low abundance can negatively alter immune response in the gut (24, 25).

Characterization of *Akkermansia* LOS in the type strain, *Akkermansia muciniphila* Muc^T^, revealed a surprising diversity of structures, including hexa-, penta-, and tetra-acylated lipid A (26). The LOS was observed to signal *via* both TLR4 and TLR2, revealing distinct signaling compared to classic interactions between LPS and TLR4 (26). Beyond this type strain, the composition of *Akkermansia* LOS is not well characterized. However, different *Akkermansia* species vary in their ability to activate TLR receptors (12), suggesting species or strain-specific structural variations in their LPS. In this study, we utilized mass spectrometry approaches to gain insight into this variation and its regulation.

## MATERIALS AND METHODS

### Bacterial strains and growth conditions

*Akkermansia* strains used in this study are listed in Table S1. *A. muciniphila* Muc^T^ (27) was obtained from ATCC. *A. ignis* strain MmAkk2 (28), *A. durhamii* RCC_PD12 (11), and all other strains have been described previously (12). Cultures were grown in mucin medium (27), which consisted of 3 mM KH_2_PO_4_, 3 mM Na_2_PO_4_, 5.6 mM NH_4_Cl, 1 mM MgCl_2_, 1 mM Na_2_S·9H_2_O, 47 mM NaHCO_3_, 1 mM CaCl_2_, 40 mM HCl, trace elements and vitamins (29), and 0.25% porcine gastric mucin (Type III, Sigma–Aldrich, St. Louis, MO, USA). Cultures were also grown in synthetic medium where mucin was replaced with 0.2% GlcNAc, 0.2% glucose, 16 g/L of soy peptone (VWR, Mississauga, ON, CA), and 4 g/L L-threonine (5). Media were supplemented with 1 mM L-cysteine (Sigma–Aldrich) and 5% vitamin K1-hemin (BD BBL, Mississauga, ON, CA) when required (i.e., for strains auxotrophic for these nutrients). All cultures were grown at 37°C in a Coy anaerobic chamber with 5% hydrogen, 5% carbon dioxide, and 90% nitrogen.

For lipid A and proteomics analyses, starter cultures were grown for 48 h and sub-cultured 1:25 into 20 mL fresh mucin medium (three biological replicates per condition). Cultures were harvested at both early logarithmic and early stationary growth phases as determined by OD_600_. At each time point, cultures were pelleted by centrifugation (13,800 × *g*, 5 min, 4°C) and stored at −80°C until analysis. For lipid A analysis, pellets from 500 µL of bacterial culture were used. For proteomics analysis, 10 mL (early logarithmic) or 5 mL (early stationary) replicates were used.

For colistin sensitivity testing, starter cultures were grown to saturation in either mucin medium or synthetic medium. The cultures were then sub-cultured 1:25 into fresh media containing 0, 0.1, 0.25, and 0.5 μg/mL of colistin (Ambeed, Buffalo Grove, IL, USA) in triplicate (300 μL total volume). Cells were collected at three predefined time points, corresponding to early logarithmic, mid logarithmic, and early stationary phases of growth, as determined by their optical density at 600 nm (OD_600_; Fig. S1). Cells were then pelleted by centrifugation at 13,800 × *g* for 5 min at 4°C and stored at −80°C until analysis.

### Whole-genome sequencing and assembly

*Akkermansia* genomic DNA was extracted using the DNeasy Blood & Tissue Kit (Qiagen) according to the manufacturer’s protocol. Long-read whole-genome sequencing and assembly were performed by Plasmidsaurus (Eugene, OR) using Oxford Nanopore Technologies. *De novo* assembly was performed using a consensus of Flye v2.9.6+ and Hifiasm (30, 31). Additional processing was performed using open-source tools hosted on GitHub, including Filtlong v0.2.1 for read filtering, Autocycler for consensus generation, dnaapler for origin rotation, and Medaka v1.8.0 for polishing. The final assemblies for isolates Akk1813 and Akk1410 achieved a mean coverage of 105× and 91×, respectively. Taxonomic assignment was determined by calculating average nucleotide identity (ANI) against known representative *Akkermansia* species using the PyANI module within the Anvi’o platform (v8) as previously described (12, 32).

### Lipid A analysis by matrix-assisted laser-desorption/ionization mass spectrometry

#### Lipid A extraction

All solvents used were purchased at the highest purity possible (HPLC grade, Fisher Scientific, Waltham, MA, USA). Lipid A was prepared using the Fast Lipid Analysis Technique (FLAT), as described by Sorensen et al. (33) with minor modifications. Briefly, *Akkermansia* pellets were thawed and resuspended in 50 μL of water (Fisher Scientific). Two microliters of sample were spotted onto a 96-well steel MALDI target (MFX μFocus plate 2,600 μm; HST Inc., South Korea) and air dried at room temperature. Two microliters of extraction buffer (0.2 M citric acid, 0.1 M trisodium citrate) was spotted, and samples were incubated at 121°C for 60 min in a humidified chamber. Samples were allowed to cool to room temperature before being gently rinsed for at least 20 s with water. Samples were then dried at 30°C before being overlaid with 2 μL of norharmane matrix (7 mg/mL in 2:1 chloroform:methanol; Sigma-Aldrich).

#### Mass spectrometry analysis

Mass spectrometry analysis was performed on a Bruker timsTOF fleX MALDI-2 instrument (Bruker Daltonics, Bremen, Germany) in the negative ion mode. Mass spectra were collected over an *m/z* range of 600–4,000 using a Nd:YAG smartbeam 3D laser at 5,000 Hz. For each sample, 1,000 laser shots were accumulated at a laser intensity of 65%. The instrument was externally calibrated with ESI-L Low Concentration Tuning Mix (Agilent Technologies Inc., Santa Clara, CA, USA). Data were acquired using timsControl 6 (Bruker Daltonics).

#### Tandem mass spectrometry analysis

Fragmentation analysis of selected lipid A ions was performed on the same instrument in MS/MS mode. Precursor ions corresponding to dominant unmodified and pEtN-modified lipid A species were isolated with a window of ±1.0 *m/z* and fragmented by collision-induced dissociation (CID) at a collision energy of 110 eV. Fragment ions were acquired over an *m/z* scan range of 200–2,500 with 5,000 shots per spectrum. Fragment ions were assigned by manual interpretation of diagnostic cleavages and by comparison with theoretical lipid A fragmentation patterns.

### Proteomics analyses by liquid chromatography tandem mass spectrometry

#### Protein extraction, digestion, and peptide clean up

All solvents used were purchased at the highest purity possible (HPLC grade, Fisher Scientific). Bacterial pellets were thawed on ice, and residual supernatant was removed by centrifugation at 2,000 × *g* for 5 min at 4°C. Pellets were resuspended in 1 mL lysis buffer (4.5 M urea and 150 mM Tris, pH 8.0) and disrupted with brief cycles of probe sonication on ice until all visible cellular debris disappeared. Proteins were precipitated with four volumes of ice-cold acetone, and samples were incubated overnight at −20°C. Samples were then centrifuged at 6,000 × *g* for 10 min at 4°C, and the resulting supernatant was removed. Pellets were resuspended in buffer (9 M urea and 300 mM Tris, pH 8.0), and protein concentration was determined by the Bradford assay using bovine serum albumin (BSA) standards in a 96-well plate format (Corning, Corning, NY, USA) using a Varioskan LUX (Thermo Scientific, Waltham, MA, USA). For in-solution digestion, 20 μg of total protein per sample was transferred to a new low-bind tube (Eppendorf, Hamburg, Germany; Cat # N217632P) and adjusted to 40 μL with 9 M urea and 300 mM Tris (pH 8.0). Disulfide bonds were reduced by the addition of dithiothreitol (DTT, Sigma-Aldrich) to a final concentration of 20 mM, followed by incubation at 37°C for 30 min. Samples were alkylated with iodoacetamide (IAA, Sigma-Aldrich) at a final concentration of 40 mM for 30 min at room temperature in the dark. Following reduction and alkylation, samples were diluted to 100 mM Tris (pH 8.0). Sequencing-grade trypsin (Promega, Madison, WI, USA) was added at a 50:1 protein to enzyme ratio (wt/wt), and samples were incubated at 37°C for 18 h. Peptides were acidified to 1% (vol/vol) formic acid, desalted using Pierce C18 tips (100 μL bed volume, Thermo Fisher Scientific), and equilibrated with 50% acetonitrile, followed by the addition of formic acid to 0.1% final volume. Bound peptides were washed with 5% acetonitrile with 0.1% formic acid and eluted with 70% acetonitrile and 0.1% formic acid into fresh low-bind tubes. Samples were then dried under a stream of nitrogen gas at 35°C and stored at 4°C until downstream analysis.

#### Peptide desalting and Evotip loading

Further peptide desalting was carried out using Evotip Pure C18 tips (Evosep, Odense, Denmark), following the manufacturer’s instructions with slightly adjusted volume for tip loading (i.e., 150 μL of solvent A instead of 200 μL; see below). Prior to sample loading, each tip was activated by adding 20 μL of buffer B (0.1% formic acid in acetonitrile) and centrifuging at 800 × *g* for 1 min. The C18 material was then wetted by briefly immersing the tips in 100% 2-propanol for approximately 10 s. For equilibration, 20 µL of buffer A (0.1% formic acid in water) was added and the tips were spun again for 60 s at 800 × *g*. Approximately 300 ng of peptide digest was applied to each tip and centrifuged to allow binding. Bound peptides were washed with an additional 20 µL of buffer A, and to maintain hydration, 150 µL of buffer A was added before a 10 s spin at 800 × *g*. Tips were kept wet until LC-MS/MS injection.

#### Mass spectrometry (LC-MS/MS)

Peptide separation and mass spectrometric analysis were performed using an Evosep One system (Evosep Biosystems, Odense, Denmark) interfaced with an Orbitrap Fusion Tribrid mass spectrometer (Thermo Scientific, Waltham, MA, USA) operated with a Nanospray Flex NG source. Chromatographic separation employed an Endurance column (EV1106; ReproSil-Pur C18, 1.9 μm, Dr. Maisch; 15 cm × 150 µm, Evosep Biosystems, Odense, Denmark). Samples were analyzed using the 15-samples-per-day extended gradient, producing a total cycle time of approximately 93 min. Peptides were introduced through a 30 µm ID stainless steel emitter (EV1086, Evosep Biosystems, Odense, Denmark). Column conditioning was performed at 1,500 nL/min, followed by the analytical gradient at 220 nL/min for 88 min, after which flow was increased again to 1,500 nL/min for column washing. Data were acquired in positive-ion mode under a DDA regime with a 3 s cycle. MS1 scans were collected in the Orbitrap at 60,000 resolution across *m/z* 350–1,800, using an AGC target of 4.0 × 10^5^ and a 118 ms maximum injection time. Precursor ions (charge states 2+ to 5+) were isolated with an *m/z* 1.6 quadrupole window and fragmented using HCD with stepped collision energies of 28%, 30%, and 32%. MS/MS spectra were recorded in the ion trap at rapid scan speed. Dynamic exclusion was set to 7.5 s with a repeat count of 2 and a mass tolerance of ±10 ppm. Lock mass correction was applied using the polysiloxane ion at *m/z* 445.12002.

#### Protein identification, quantification, and filtering

Raw LC-MS/MS data were processed using the FragPipe computational platform (version 23.1), using MSFragger (version 4.3) for peptide-spectrum matching (34) and IonQuant (version 1.11.11) for quantitative analysis (35). Searches were performed against three custom *A. muciniphila* databases, combined with common contaminants and decoys. MSFragger searches used a ±10 ppm precursor tolerance and a 20 ppm fragment tolerance. Trypsin specificity was applied with up to two missed cleavages. Carbamidomethylation of cysteine (Cys) induced by IAA was included as a fixed modification, while methionine (Met) oxidation and protein N-terminal acetylation were allowed as variable modifications. Peptide spectrum matches were rescored using Percolator (36), and protein identifications were filtered to a 1% FDR via ProteinProphet (37). Label-free quantification was conducted with IonQuant, using the MaxLFQ algorithm (38) with match-between-runs enabled.

### Data and statistical analysis

#### Lipidomics

Mass spectra and tandem mass spectra were processed in DataAnalysis 6.1 (Bruker Daltonics). Statistical tests were performed using GraphPad Prism v10 (GraphPad Software, Boston, MA, USA). Figures were generated in GraphPad Prism v10 and Adobe Illustrator (Adobe, San Jose, CA, USA).

#### Proteomics

Raw proteomics files were uploaded to FragPipe Analyst to impute and log-transform the MaxLFQ data. The resulting CSV from this process was downloaded and used for subsequent data analysis. To evaluate sample reproducibility and variance across experimental groups, a principal component analysis (PCA) was completed in an R statistical environment (39, 40) using the prcomp function and ggplot2 for plotting the resulting dimensionally-reduced matrix. Perseus was used to classify data into the experimental groups. Differences in protein expression between experimental groups were calculated and expressed as a Log2 Fold Change (Log2 FC). Statistical analysis was performed using a two-way ANOVA with post-hoc pairwise comparisons and the Benjamini-Hochberg (BH) adjustment for multiple comparisons using the emmeans package in R. Differences in protein abundance were considered statistically significantly if the BH-adjusted *P* value was <0.05 and had the absolute value of Log2 FC was ≥1.

## RESULTS AND DISCUSSION

### Lipid A profiling reveals phosphoethanolamine modifications in *Akkermansia*

Altogether, 27 *A. muciniphila*, 19 *A. massiliensis*, 7 *A. biwaensis*, and 1 isolate of each *A. ignis* and *A. durhamii* were included in the initial screening (Table S1). Isolates were sourced from diverse clinical and experimental contexts, including pediatric obesity, healthy weight controls, cancer immunotherapy, and murine colitis cohorts, to maximize species coverage and genomic diversity (11, 28, 41). All isolates were derived from humans, except for *A. ignis*, which was isolated from a mouse (28). The strains were cultured in porcine gastric mucin medium to early stationary phase, and their lipid A profiles were established using MALDI mass spectrometry following the Fast Lipid Analysis Technique (FLAT) (33).

**Fig 1:**
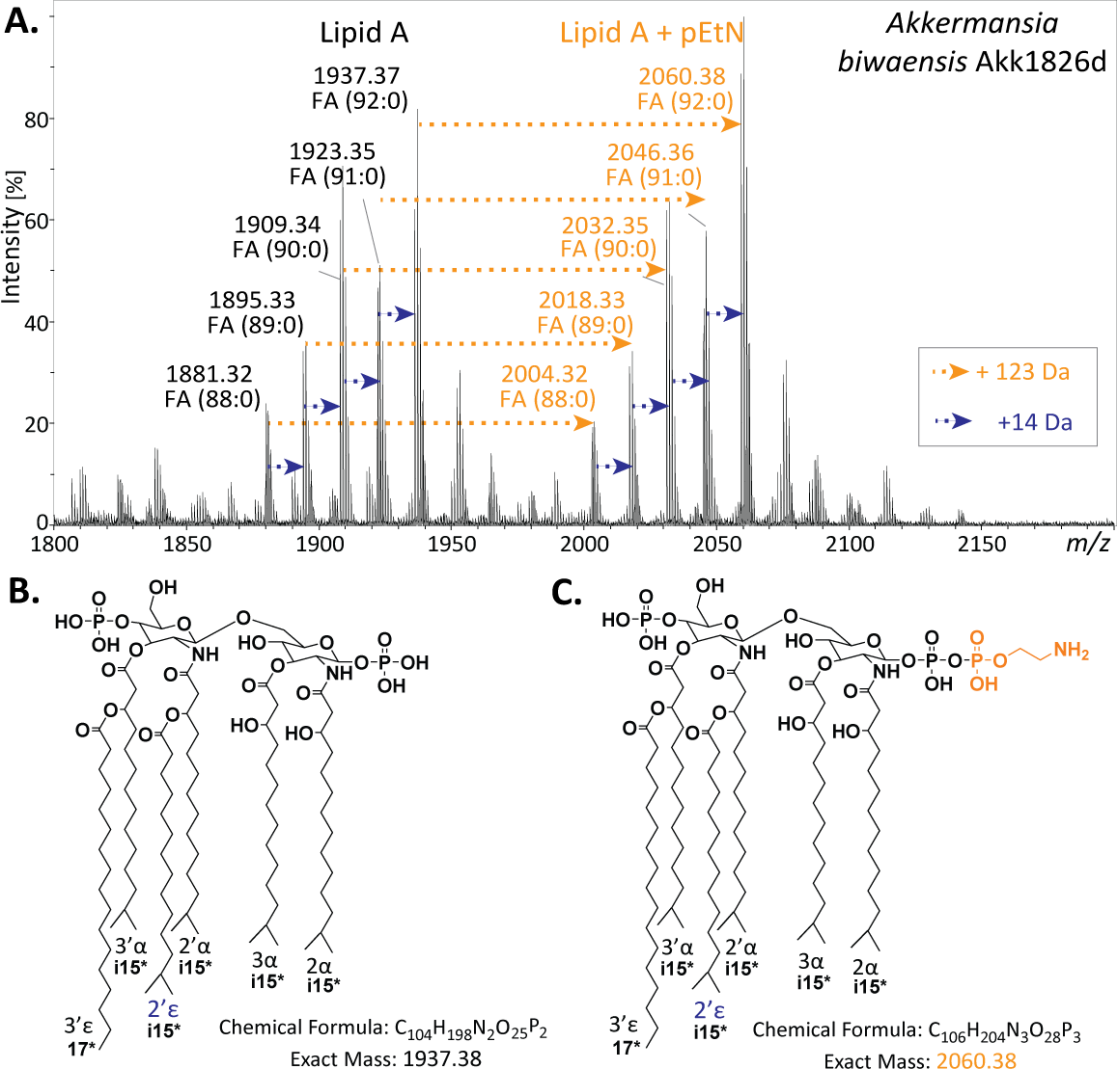
Lipid A profile of representative species, *A. biwaensis* isolate Akk1826d, as determined by MALDI MS. (**A**). Annotated lipid A profile. Orange arrows represent an addition of the pEtN modification to the signature lipid A molecule (+123 Da). Numbers in brackets represent the total number of carbons and double bonds in all fatty acid (FA) chains in individual lipid A molecules. Blue arrows represent a one-carbon difference in length of the fatty acyl chains at the 2’ε position that drives the lipid A structural diversity within the isolate (+14 Da; CH_2_). (**B**) Proposed structure of the signature, non-modified lipid A. (**C**) Proposed structure of the modified lipid A. Asterisks denote possible branching of fatty acyl chains based on previous reports (26); these are not detectable by mass spectrometry approaches used here. Data were collected in the negative ion mode on a timsTOF fleX MALDI-2 instrument. Representative tandem mass spectra can be found in the supplementary materials (Fig. S1).

Two forms of lipid A were detected in all isolates. The first form was identical to that previously described in *Akkermansia* by Garcia-Vello et al., which was characterized using a set of standardized methods for lipid A structural elucidation (25). It consisted of hexa-acylated, di-phosphorylated lipid A species with a glucosamine disaccharide backbone (Fig. 1A, black label, and Fig. 1B). The second form consisted of lipid A species modified by the addition of phosphoethanolamine (pEtN) to the 1-terminal phosphate (Fig. 1A, orange label, and Fig. 1C). The modification is routinely detected as a mass shift of *m/z* 123 greater than the signature ion (32), and its presence at the 1-terminal (C1) phosphate was confirmed by tandem mass spectrometry (Fig. 1C; Fig. S2). Although *Akkermansia* LOS has been recently analyzed in detail, the pEtN modification has not been reported. It is possible that its presence may have been previously missed due to the lower scan range utilized in the cited study (scan range up to *m/z* 2,000 and *m/z* 4,000 were investigated by Garcia-Vello et al. [26] and the present study, respectively). Penta- and tetra-acylated lipid A species reported by Vello et al. were also present, albeit at relatively low abundance.

**Fig 2:**
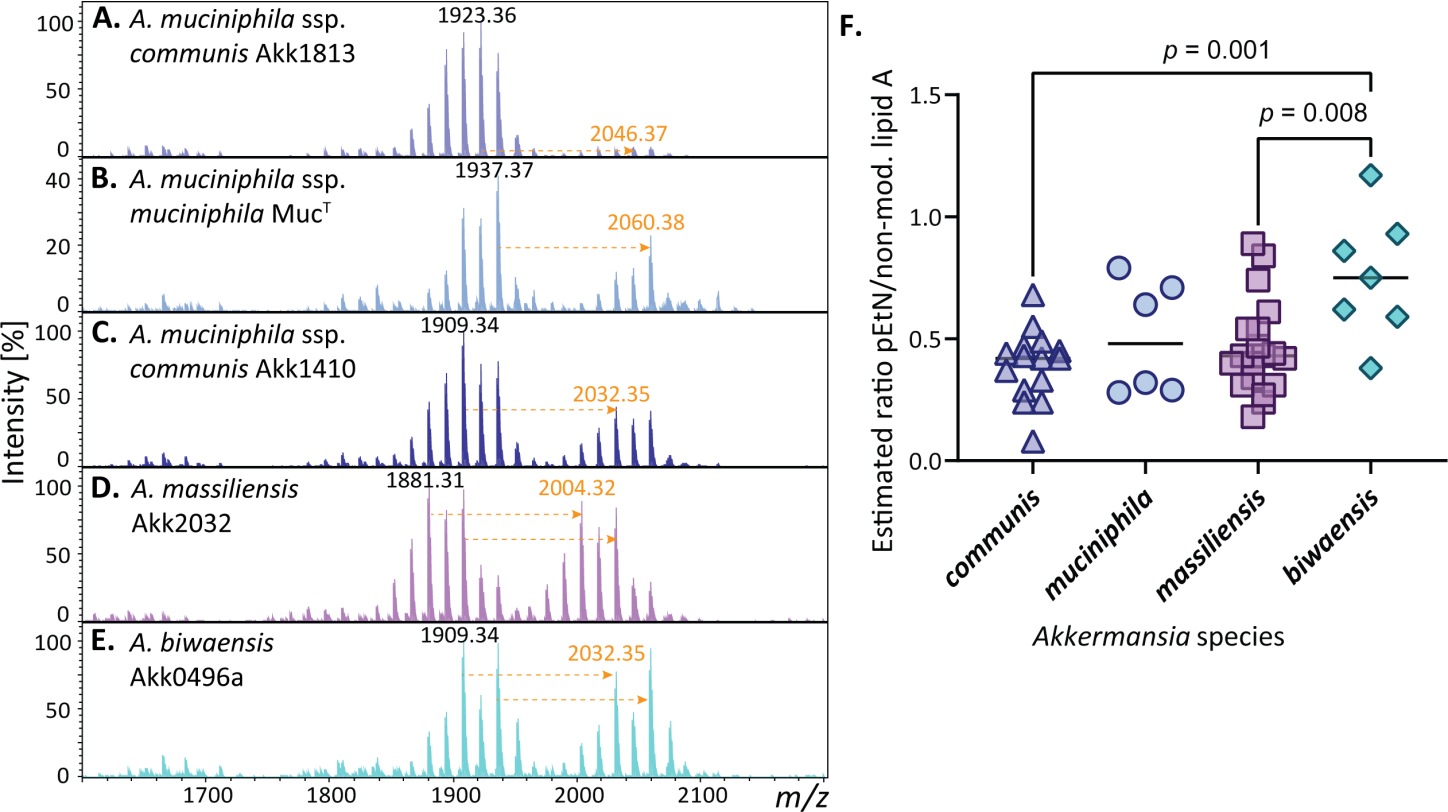
Lipid A profiles of representative isolates with different relative ratio of pEtN modified to non-modified lipid. (**A**) *A. muciniphila* ssp. *communis* Akk1813. (**B**) Type strain *A. muciniphila* ssp. *muciniphila* Muc^T^. (**C**) *A. muciniphila* ssp. *communis* Akk1410. (**D**) *A. massiliensis* Akk2032. (**E**) *A. biwaensis* Akk0496a. Data were collected in the negative ion mode on a timsTOF *flex* MALDI-2 instrument. Representative mass spectra from three biological replicates grown to the stationary phase in mucin medium are shown. Black labels: non-modified lipid, orange labels: pEtN-modified lipid A. (**F**) Ratio of pEtN-modified to non-modified lipid A across screened *Akkermansia* isolates. On the *x*-axis, *communis* and *muciniphila* refer to *A. muciniphila* subsp. *communis* and *A. muciniphila* subsp. *muciniphila*, respectively. *P* values determined by one-way ANOVA with Tukey’s post-hoc test.

Lipid A is often used as a molecular fingerprint, aiding in bacterial identification, characterization, and diagnostics of infections caused by diderm pathogens (33, 42, 43). Across all *Akkermansia* isolates, diverse lipid A profiles were identified, and they largely corresponded to specific *Akkermansia* species and subspecies (Fig. 2). At early stationary phase, most *A. muciniphila* isolates contained a signature, non-modified lipid A ion with *m/z* 1,909.34 or 1,923.36 (Fig. 2A through C; Fig. S3). Most *A. massiliensis* isolates contained two signature ions of comparable abundance, *m/z* 1,881.31 and 1,909.34 (Fig. 2D; Fig. S4). Finally, most *A. biwaensis* isolates also contained two signature ions with similar abundance, *m/z* 1,909.34 and 1,937.37 (Fig. 2E; Fig. S5). Therefore, if used for bacterial identification, signature ions with *m/z* values of 1,881.31, 1,909.34, 1,923.36, and 1,937.37 are characteristic of the *Akkermansia* species analyzed here (Fig. 2; Fig. S3 to S5). Because MALDI MS lipid A profiles for taxonomically related bacteria within the *Verrucomicrobiota* phylum are currently lacking in the literature, future comparative profiling will be necessary to determine if these signatures serve as unique diagnostic biomarkers for the *Akkermansia* genus. Additional structural diversity was detected within each isolate, likely caused by the variable length of the secondary fatty acyl chain at the 2’ε position (Fig. 1; Fig. S2). This variation in fatty acid chain length was deduced from consistent mass shifts of 14 Da in our MALDI MS and MS/MS spectra (Fig. 1; Fig. S2).

The ratios between the pEtN-modified and non-modified lipid A signature ions were then established; these ratios varied between isolates. Overall, the *A. muciniphila* isolate Akk1813 had the lowest ratio (Fig. 2A). In contrast, *A. biwaensis* isolate Akk1826d and *A. massiliensis* isolate Akk2190 had the highest ratios (Fig. 1A; Table S1). On average, the highest relative abundance of pEtN-lipid A was found in *A. biwaensis* isolates (Fig. 2F).

### Impact of pEtN lipid A modification on sensitivity to the antimicrobial peptide colistin

**Fig 3:**
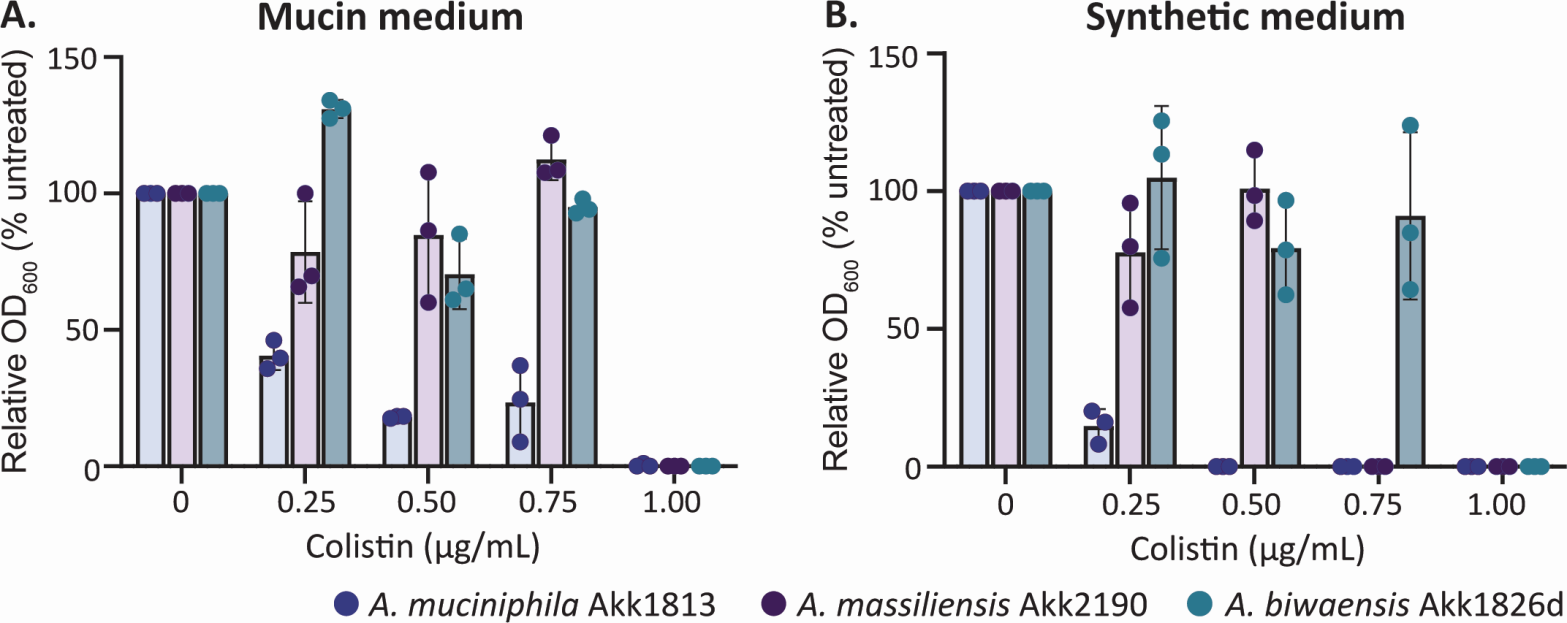
Colistin susceptibility of representative *Akkermansia* isolates with varying abundance of pEtN-modified lipid A in mucin and synthetic media. (**A**) Mucin medium. (**B**) Synthetic medium. Growth was monitored by optical density (OD_600_) measurements and normalized to untreated controls. Data represent mean ± SD of three biological replicates.

The addition of pEtN to lipid A is associated with resistance to antimicrobial peptides (44). To investigate the function of the pEtN modification in *Akkermansia*, three representative isolates were grown in increasing concentrations of colistin (0, 0.1, 0.25, and 5 µg/mL). The experiment was performed in both mucin medium, which most closely resembles the physiological niche of *Akkermansia*, and synthetic medium, which has been shown to produce higher levels of TLR4/MD2 activation compared to mucin-grown cells (12). Two trends were observed. First, isolates with a higher ratio of pEtN-lipid A to lipid A were less sensitive to colistin. For example, the *A. biwaensis* isolate Akk1826d, which has a high relative abundance of pEtN-lipid A (Fig. 1), was able to grow in higher concentrations of colistin than the *A. muciniphila* isolate Akk1813 containing low levels of pEtN-lipid A (Fig. 2A). Second, isolates Akk1813 and Akk2190 displayed lower sensitivity to colistin in mucin medium (Fig. 3A) when compared to synthetic medium (Fig. 3B). The function of the modification is most extensively studied in diderm pathogens. In obligate gut commensals, it has also been described to decrease sensitivity to antibiotics, including colistin and polymyxin B, in *Escherichia coli* strain Nissle 1917 (45).

### Effect of growth phase and culturing media on pEtN modification of lipid A

**Fig 4:**
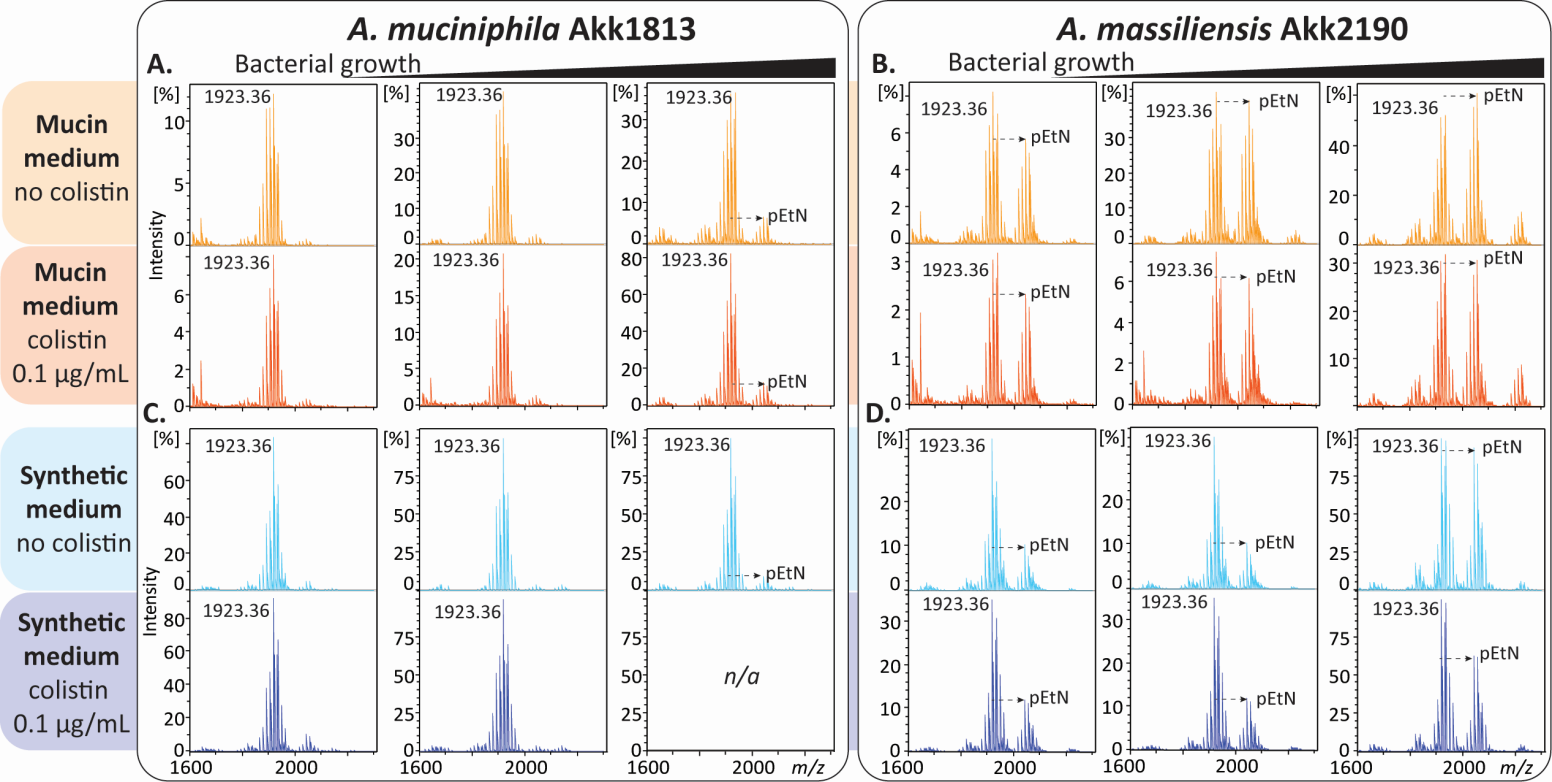
Lipid A profiles of representative *Akkermansia* isolates with varying abundance of pEtN-modified lipid A in different growth conditions. (**A and B**) Mucin medium. (**C and D**) Synthetic medium. (**A and C**) *A. muciniphila* Akk1813 (low pEtN abundance). (**B and D**) *A. massiliensis* Akk2190 (high pEtN abundance). Samples were taken at the early- and mid-logarithmic and in early stationary phases. Corresponding mass spectra are organized according to the increasing optical density increases from left to right (low to high). *n/a*—cells were not viable at the specified growth condition. Data were collected in the negative ion mode on a timsTOF *flex* MALDI-2 instrument. Representative spectra from three biological replicates are shown. A minor ion cluster around *m/z* 2100 was detected in the lipid A profile of the Akk2190 strain during the stationary phase (panel **B**). This cluster corresponds to norharmane matrix adducts, as determined by tandem mass spectrometry.

To investigate the sensitivity to colistin in different growth media further, lipid A profiles of *A. muciniphila* Akk1813 and *A. massiliensis* Akk2190 were characterized in the corresponding growth conditions. The highly susceptible Akk1813 isolate (Fig. 3) displayed consistently low relative abundance of pEtN-lipid A (Fig. 4A and C). In contrast, the Akk2190 isolate displayed higher relative abundance of pEtN-lipid A in mucin medium (Fig. 4B) when compared with the synthetic medium (Fig. 4D). This was in line with the colistin susceptibility testing (Fig. 3) and is indicative of a protective role of the pEtN modification against the antimicrobial peptide. The modification is also associated with reduced endotoxicity of lipid A, as it leads to decreased binding to the TLR4/MD2 complex, dampening the downstream inflammatory cascades (46). Higher abundance of the pEtN-lipid A might also be contributing to the decreased activation of TRL4/MD2 by *Akkermansia* grown in the mucin medium when compared to synthetic medium (12).

Intriguingly, the ratio between pEtN-modified and non-modified lipid A was unexpectedly dynamic in the Akk2190 isolate. It increased as the cells reached higher optical density even in the absence of colistin (Fig. 4B and D). This trend was confirmed using two other *Akkermansia* isolates, the *A. muciniphila* type strain Muc^T^ (Fig. S6A) and Akk0500a (Fig. S6B). Surprisingly, this observation suggests that pEtN modification of lipid A is not induced by the presence of antimicrobial peptides but rather increases as *Akkermansia* reaches later stages of growth. The stationary phase is associated with limited nutrient availability and, eventually, starvation. In *E. coli*, starvation induces stress response pathways that lead to the expression of lipid A-modifying enzymes (47). It is possible that similar defense mechanisms are present in *Akkermansia*.

### Putative pEtN transferase in *Akkermansia*

Phosphoethanolamine modifications to lipid A are carried out by pEtN transferases (33). These enzymes are often carried on mobile elements, such as *mcr* plasmids (48), which have not been identified in any *Akkermansia* genomes (11, 12, 28). Therefore, we used AlphaFold Foldseek (49) to search the reference *Akkermansia* proteome against EptA, a chromosomally encoded pEtN transferase. Although annotated as a sulfatase, the B2UQG2_AKKM8 protein (UniProt ID), encoded by *amuc*_*0864* in *A. muciniphila* Muc^T^, was identified as the closest match. It contains a pEtN transferase domain at its N-terminus (79–183 aa; based on UniProt annotation), making it the strongest candidate identified in this analysis. However, a transposon mutant in the corresponding gene was not available in our existing library (50), and we were unable to experimentally confirm its function.

Multiple sequence alignment did not reveal any amino acid substitutions in the predicted functional domain that were present exclusively in the Akk1813 isolate, which exhibited extremely low relative abundance of pEtN-lipid A. In addition, the corresponding RNA levels did not differ between *Akkermansia* grown in synthetic and mucin media (50). Therefore, even if *amuc_0864* encodes an *Akkermansia* pEtN transferase, additional layers of regulation are likely responsible for the observed phenotypic differences between individual isolates.

### Proteomics analysis of cell envelope remodeling pathways across different growth stages

To gain additional insights into the regulation of pEtN modification, we focused on other proteins and pathways associated with cell envelope remodeling in diderm bacteria. Specifically, we compared protein expression of the *A. muciniphila* ssp. *communis* Akk1813 isolate, which was unable to increase the relative abundance of pEtN-modified lipid A in the later stages of growth (Fig. 4A and C), with *A. muciniphila* ssp. *muciniphila* isolates Muc^T^ and Akk0500a that were able to do so (Fig. S6). Cells were grown in mucin medium and harvested at early logarithmic and early stationary phases (OD_600_ of 0.03 ± 0.02 and 0.24 ± 0.03, respectively; average ± standard deviation; *N* = 3) (Fig. S1). In this study, 1,408, 1,532, and 1,470 proteins were identified in Akk1813, Muc^T^, and Akk0500a, respectively (Fig. S7A). Principal component analysis (PCA) showed that biological replicates clustered together, with samples separating primarily by growth stage along PC1 (25.8% variance) and by isolate type and subspecies along PC2 (23.0% variance) (Fig. S7B).

Lipid A pEtN modification occurs in the periplasmic space before the molecule is flipped to the outer membrane (51). Therefore, a bacterium must produce lipid A *de novo* to modify it. Lipid A is synthesized by a conserved Raetz pathway (Table S2), and the individual proteins displayed no changes in their abundance between the logarithmic and stationary phases of growth (Fig. S8). The first committed step of the pathway is catalyzed by LpxC and tightly regulated in diderm bacteria, translating into regulation of LPS (or LOS) synthesis (52). The abundance of the LpxC protein was the lowest the Akk1813 strain when compared to Muc^T^ and Akk0500a albeit the trend was not statistically significant (Fig. S8C).

**Fig 5:**
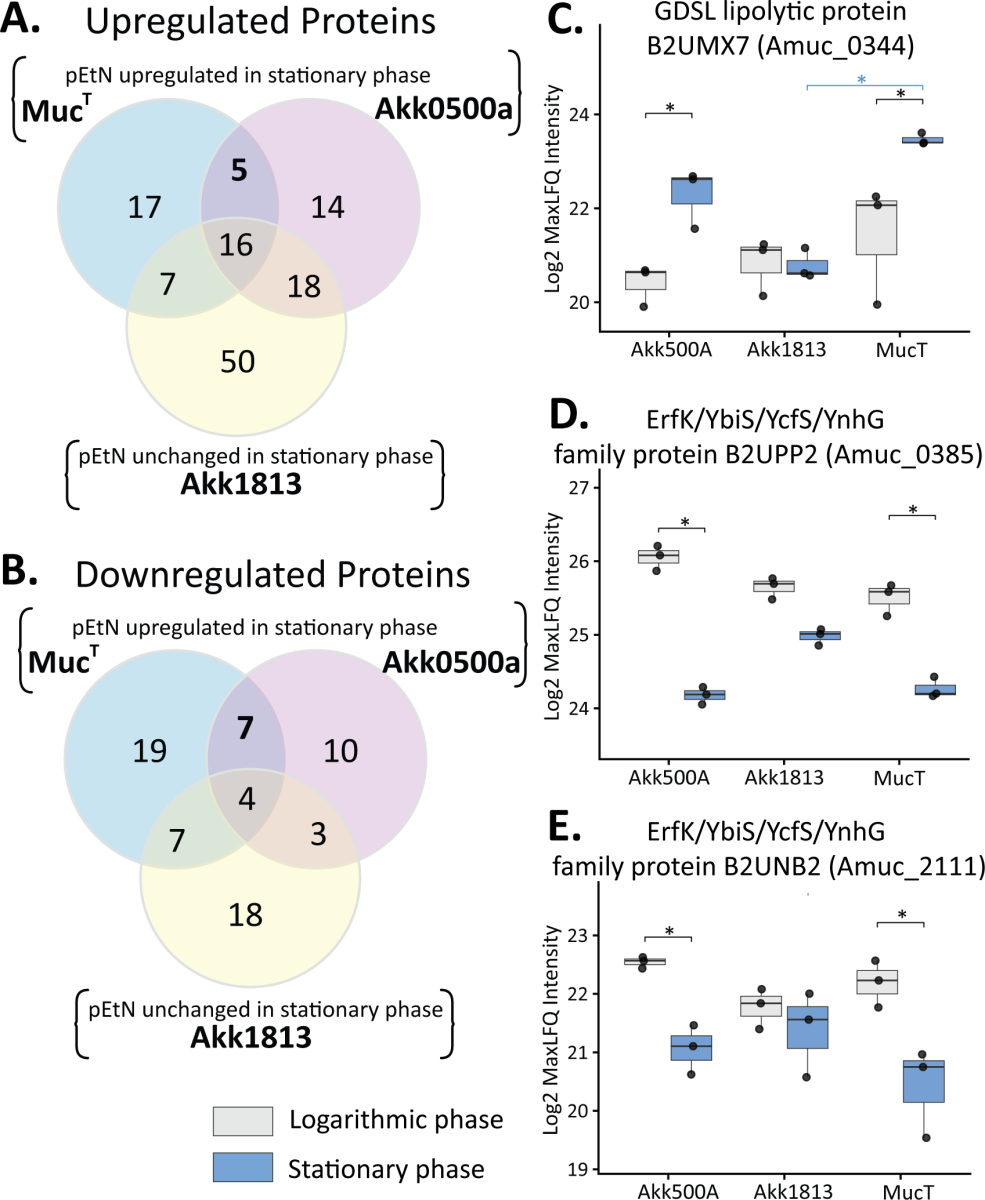
Differential protein abundance associated with growth phase and pEtN-modification phenotypes in *Akkermansia muciniphila*. (**A and B**) Venn diagrams showing the number of significantly (**A**) upregulated and (**B**) downregulated proteins exclusively in Muc^T^ and Akk0500a (isolates with high pEtN in stationary phase) compared to Akk1813 (low/unchanged pEtN). (**C–E**) Log2 MaxLFQ intensities of representative differentially abundant proteins: (**C**) GDSL lipolytic protein B2UMX7 (Amuc_0344) which was upregulated in both phenotypic isolates, (**D**) ErfK/YbiS/YcfS/YnhG family protein B2UPP2 (Amuc_0385), and (**E**) ErfK/YbiS/YcfS/YnhG family protein B2UNB2 (Amuc_2111). For panel **C–E**, light gray boxes represent the early logarithmic phase and blue boxes represent the early stationary phase for each indicated isolate.

Next, we compared protein expression between the growth phases. For clarity, proteins and genes are referred to by their *A. muciniphila* Muc^T^ identifiers. Proteins that were significantly upregulated or downregulated exclusively in Muc^T^ and Akk0500a during the early stationary phase were extracted and annotated (Table S3; Fig. 5). In total, the abundance of 12 proteins was altered in both strains when compared to Akk1813 (5 upregulated, Fig. 5A, and 7 downregulated, Fig. 5B). Several proteins putatively associated with cell envelope synthesis, maintenance, and remodeling were revealed.

In both isolates, the expression of a GDSL lipolytic protein B2UMX7 encoded by *amuc_0344* was upregulated (Fig. 5C). An additional GDSL lipolytic protein (B2ULH8; *amuc_1544*) was upregulated in Muc^T^ only. These enzymes could contribute to lipid turnover, necessary for maintenance of outer membrane asymmetry during cell wall remodeling.

In contrast, two ErfK/YbiS/YcfS/YnhG family proteins linked to peptidoglycan crosslinking and anchoring it to the outer membrane during synthesis (53) were downregulated in both Muc^T^ and Akk0500a (B2UNB2 and B2UPP2, encoded by *amuc_0385* and *amuc_2111* genes, respectively, Fig. 5D and E). In the Muc^T^ isolate, an additional downregulated ErfK/YbiS/YcfS/YnhG protein was detected (B2UMM7; *amuc_1765*). Finally, two N-acetylmuramoyl-L-alanine amidases and a peptidoglycan-binding protein LysM, all associated with peptidoglycan turnover, were also downregulated in the Akk0500a isolate (B2URI4, B2UQK0, and B2UPV9, encoded by *amuc_1244*, *amuc_0903*, and *amuc_0657*, respectively).

There is no known direct connection between peptidoglycan turnover and the addition of functional groups to lipid A. However, the peptidoglycan and lipid A synthesis pathways compete for a sugar precursor (UDP-GlcNAc). In *Pseudomonas aeruginosa*, the pathways are linked *via* an interaction between LpxC and MurA, the first committed enzyme of the peptidoglycan synthesis pathway (54). A non-significant trend toward lower MurA abundance was observed in the early stationary phase across all three strains (Fig. S9A).

**Fig 6:**
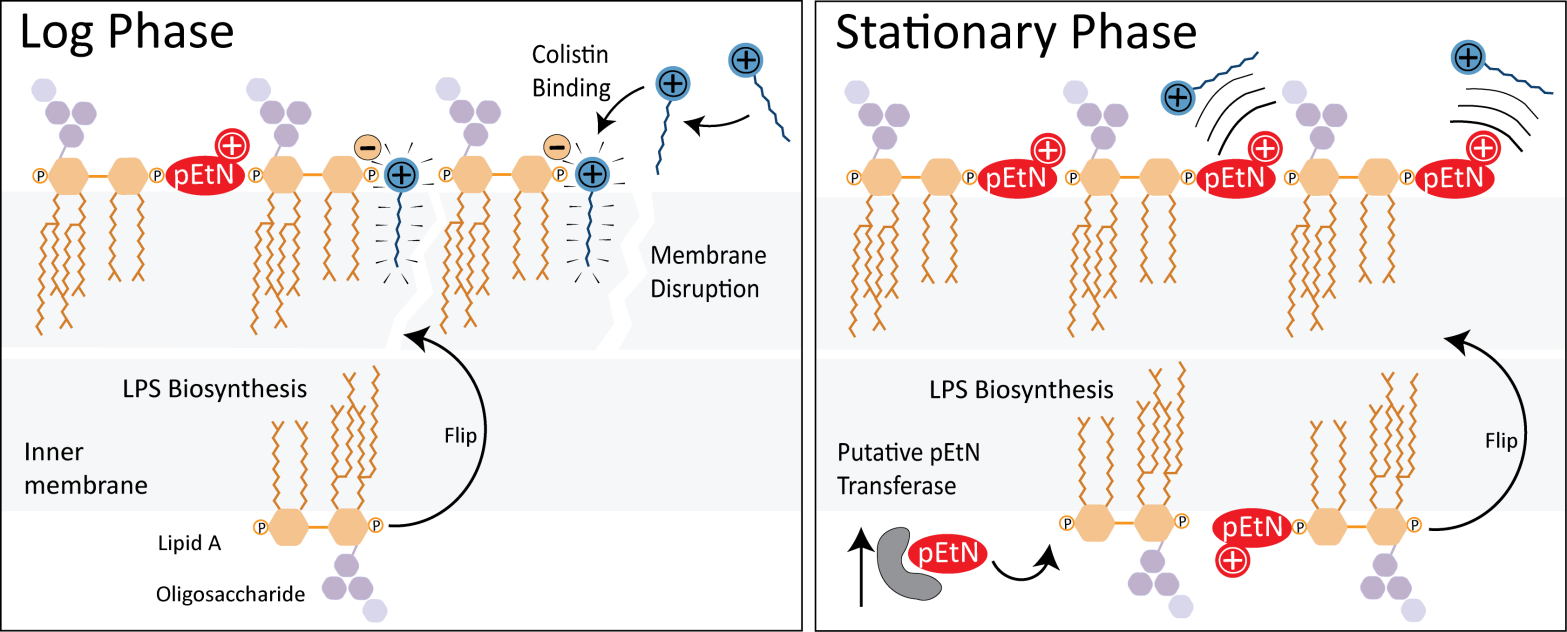
Proposed model of pEtN modification dynamics and function in *Akkermansia* lipid A.

To conclude, we discovered a pEtN modification of lipid A that was present in all screened isolates of *Akkermansia*, spanning five distinct species. Individual isolates displayed various relative abundances of the pEtN-lipid A, and higher levels were associated with lower sensitivity to the antimicrobial peptide colistin (Fig. 6). The abundance of pEtN-lipid A increased in the late stages of growth *via* yet unknown regulatory mechanisms and was possibly accompanied by membrane lipid remodeling and reduced peptidoglycan turnover. Further research on the modification and its impact on *Akkermansia* biology and host colonization is warranted.

## Data Availability

Genomic data for strains Akk1813 and Akk1410 are available under NCBI BioProject PRJNA1423308. The mass spectrometry proteomics data have been deposited to the ProteomeXchange Consortium via the PRIDE partner repository (55) with the data set identifier PXD074583.

## SUPPLEMENTAL MATERIAL

Supplemental Figures and Tables spectrum.00682-26-s0001.pdf Figures S1–S9; Tables S1 and S3

Table S2 spectrum.00682-26-s0002.xlsx Dysregulated proteins during stationary phase, found exclusively in MucT and Akk0500a isolates when compared to the Akk1813 isolate.

ASM does not own the copyrights to Supplemental Material that may be linked to, or accessed through, an article. The authors have granted ASM a non-exclusive, world-wide license to publish the Supplemental Material files. Please contact the corresponding author directly for reuse.
