# Supplementary material for: Phosphoethanolamine modification of lipid A found in five distinct Akkermansia species
Source: Microbiol Spectr. 2026 Jun 22;14(8):e00682-26. doi: 10.1128/spectrum.00682-26 (PMC13436333; doi:10.1128/spectrum.00682-26)
Supplement: Supplementary file 1 [file spectrum.00682-26-s0001.pdf]

Supplementary materials for:

Phosphoethanolamine modification of lipid A found in five distinct *Akkermansia* species

Silas Porter Crenna[1†], Arianna Lagoudakis[2†], Angela M. Jackson[1,2], Kenneth Weke[2], Georgia Tiegs[1], David R. Goodlett[1,2], Lauren E. Davey[2*], and Helena Pětrošová[1,2*]

[1]University of Victoria Genome BC Proteomics Centre, Victoria, BC, CA
[2]Department of Biochemistry and Microbiology, University of Victoria, Victoria, BC, CA

*Correspondence: Lauren Davey: laurendavey@uvic.ca & Helena Pětrošová: hpetros@uvic.ca

Table S1. ***Akkermansia*** **isolates analyzed in this study.** Lipid A group classification and ratios of pEtN modification are listed. ***a,b*** as determined in early stationary phase; ***b*** as calculated from measured intensities of signature lipid A ions (pEtN-modified to non-modified) across three biological replicates per isolate. Outliers are highlighted in bold.

Species	Strain	Estimated ratio pEtN-modified to non-modified lipid A[b]
A. muciniphila subsp. muciniphila	Muc[T] (BAA-835)	0.64
	Akk0096	0.71
	Akk0200	0.32
	Akk1713	0.29
	Akk2670	0.79
	Akk0500a	0.28
A. muciniphila subsp. communis	Akk0880	0.45
	Akk1376	0.24
	Akk14745	0.29
	Akk1570	0.42
	Akk1576	0.68
	Akk1616	0.47
	Akk1683	0.24
	Akk1756	0.44
	Akk1813	**0.08**
	Akk1990	0.33
	Akk2030	0.43
	Akk2090	0.42
	Akk2650	0.37

	Akk0500b	0.55
	Akk1410	0.49
A. muciniphila (not subtyped)	Akk1700	0.33
	Akk1820	0.38
	Akk2080	0.96
	Akk2340	0.30
	Akk2543	0.41
	Akk2640	0.43
A. massiliensis	Akk05415	0.74
	Akk1476	0.34
	Akk1573	0.42
	Akk1826b	0.43
	Akk1863	0.24
	Akk18645	0.31
	Akk1866d	0.31
	Akk1900	0.27
	Akk1906	0.40
	Akk2000	0.61
	Akk2033	0.84
	Akk21215	0.44
	Akk2180	0.43
	Akk2190	0.89
	Akk2196	0.47
	Akk2583	0.54
	Akk2680	0.54
	Akk2740	0.40
	AkkB40	0.18
A. ignis	MmAkk2	0.28
A. durhamii	RCC_12PD	0.22
A. biwaensis	Akk0196	0.93
	Akk0490a	0.62
	Akk0496a	0.86
	Akk14115	0.38
	Akk1826d	**1.17**
	Akk2633	0.75
	Akk2750	0.59

Table S2 (Excel file). List of dysregulated proteins during stationary phase, found exclusively in Muc^T and Akk0500a isolates when compared to the Akk1813 isolate. Differences between early logarithmic and early stationary phases are displayed as log-fold change (Log2FC). Please note that negative values are indicative of increase in abundance. Only statistically significant changes in abundance of proteins are listed ($p < 0.05$; adjusted for multiple comparisons, adj.), as determined by Two-Way ANOVA. Proteins highlighted in bold are putatively associated with membrane biogenesis. Color indicates significant differences in abundance in both Muc^T and Akk0500a when compared to Akk1813.

Table S3. List of lipid A and peptidoglycan biosynthesis genes in *Akkermansia* species, annotated using KEGG. proteins and genes are referred to by their *A. muciniphila* Muc^T identifiers. *Not detected in the presented proteomics dataset.

Protein	UniProt ID	Locus Tag
**Lipid A biosynthesis (Raetz pathway)**		
LpxA	B2ULY0	*amuc0180*
LpxB	B2UP91	*amuc2052*
LpxC	B2UNL5	*amuc1918*
LpxD	B2UND2	*amuc406*
LpxH/I	B2UQI2	*amuc0884*
LpxK	B2UPD5	*amuc0575*
LpxL	B2ULH5	*amuc1541*
LpxR*	B2UQC1	*amuc0823*
KdtA	B2UNU2	*amuc0474*
**Peptidoglycan biosynthesis**		
MurA	B2UNJ3	*amuc1895*
MurC	B2UPW2	*amuc0660*
MurD	B2UPV8	*amuc0656*
MurE	B2UPL3	*amuc0653*
MurF	B2UPL4	*amuc0654*
MraY	B2UPL5	*amuc0655*
MurG	B2UPW1	*amuc0659*

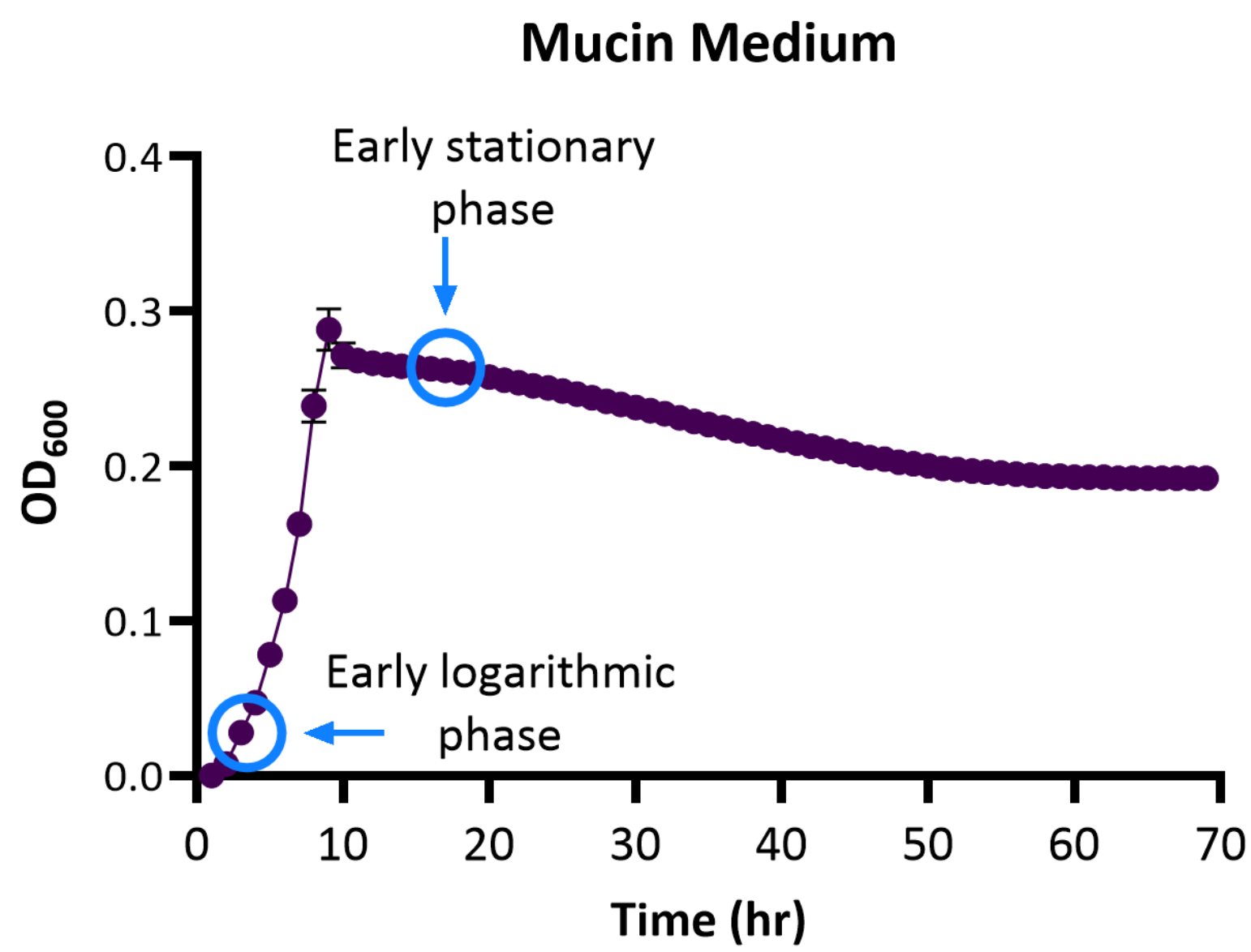

Figure S1. Growth curve of *Akkermansia muciniphila* Muc^T reference strain in mucin medium. Blue circles represent time points used for sampling in the early logarithmic and in the early stationary phases of growth. N=3 biological replicates. laurendavey@uvic.ca

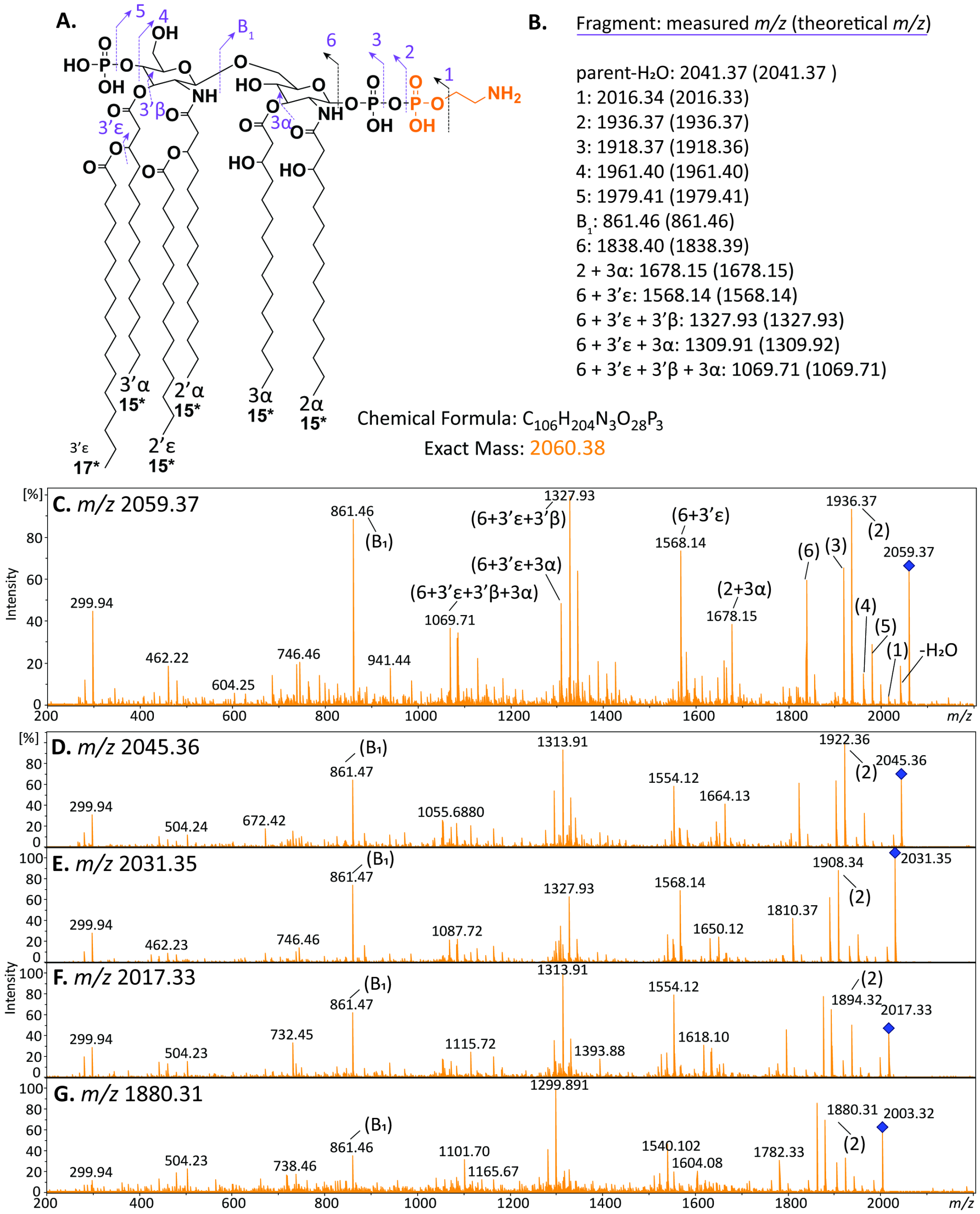

Figure S2. Tandem mass spectrometry analysis of *Akkermansia* pEtn-modified lipid A. A. Proposed structure of the signature ion of non-modified lipid A species with annotated fragmentation patterns (arrows). **B.** Mass-to-charge (*m/z*) ratios of fragment ions, as determined by tandem mass spectrometry analysis, and comparison to *m/z* of theoretical fragments, as calculated using the proposed structure. **C-G**. Annotated tandem mass spectra. **C.** Signature modified lipid A ion with *m/z* 2059.37 ($C_{106}H_{203}N_3O_{28}P_3^-$). **D.** *m/z* 2045.36 ($C_{105}H_{201}N_3O_{28}P_3^-$). **E.** *m/z* 2031.35 ($C_{104}H_{199}N_3O_{28}P_3^-$). **F.** *m/z* 2017.33 ($C_{103}H_{197}N_3O_{28}P_3^-$). **G.** *m/z* 2003.31 ($C_{102}H_{195}N_3O_{28}P_3^-$). *A. muciniphila* isolate BAA was used for analysis. Mass spectra obtained following FLAT in the negative ion mode on the timsTOF *flex* MALDI-2 (Bruker).

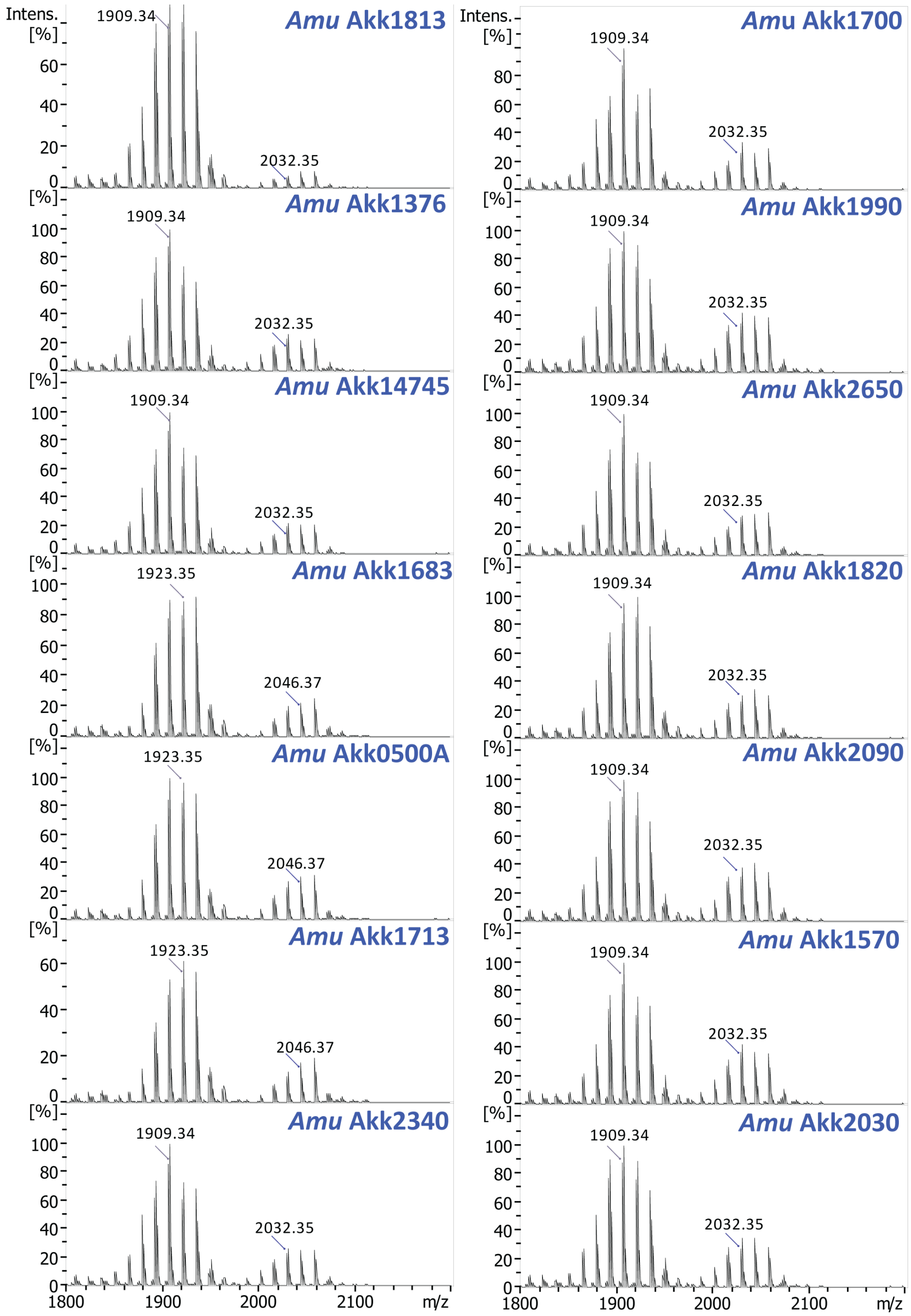

Figure S3. Lipid profiles of *Akkermansia muciniphila* (*Amu*) isolates in porcine gastric mucin medium to early stationary phase. Mass spectra obtained following FLAT in the negative ion mode on the timsTOF *flex* MALDI-2 (Bruker) from stationary phase cultures. Each mass spectrum is representative of three biological replicates; measured intensity is represented in relative units (%). Strain description can be found in **Table 1**.

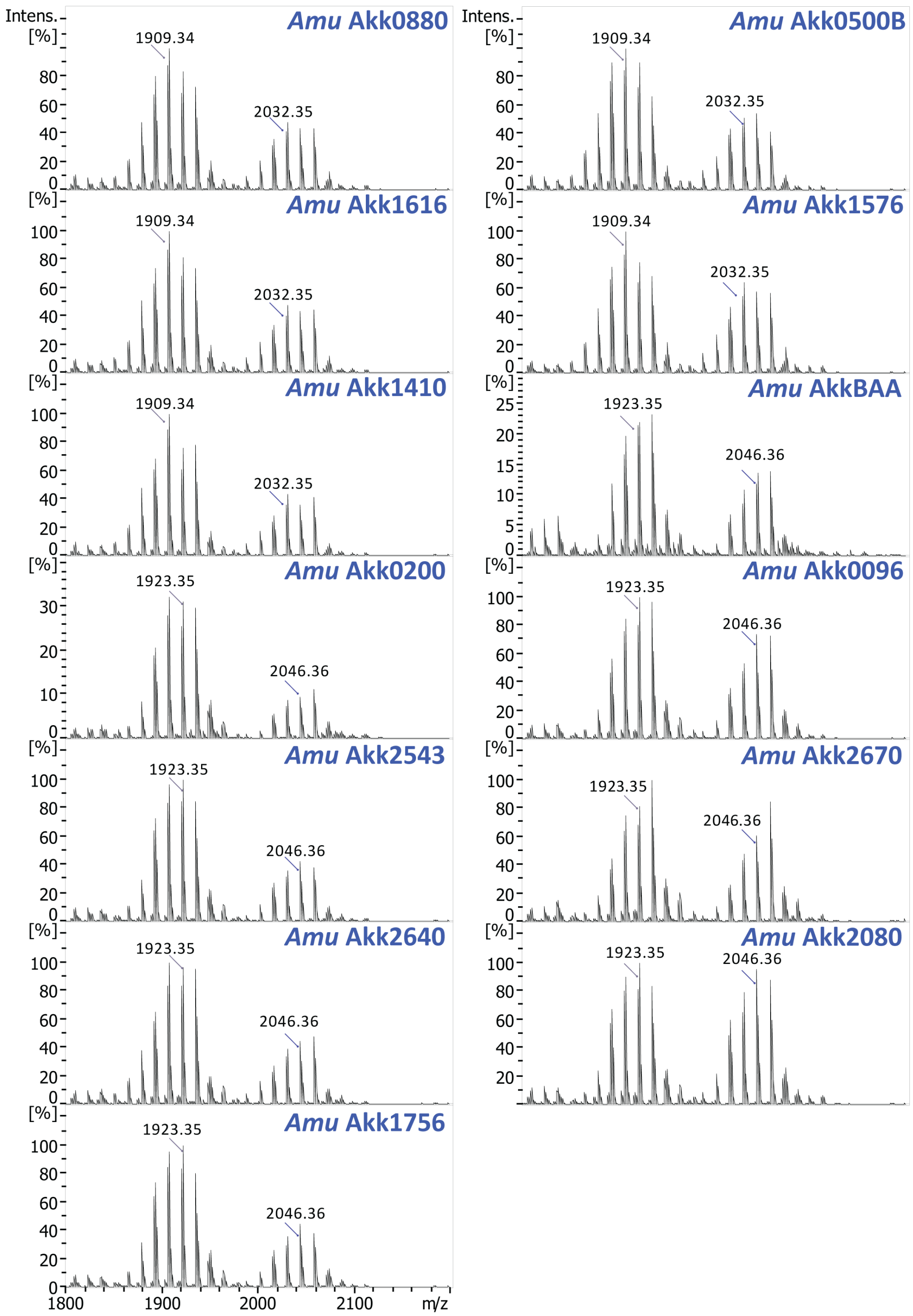

Figure S3. Lipid profiles of *Akkermansia muciniphila* (*Amu*) isolates in porcine gastric mucin medium to early stationary phase (continued).

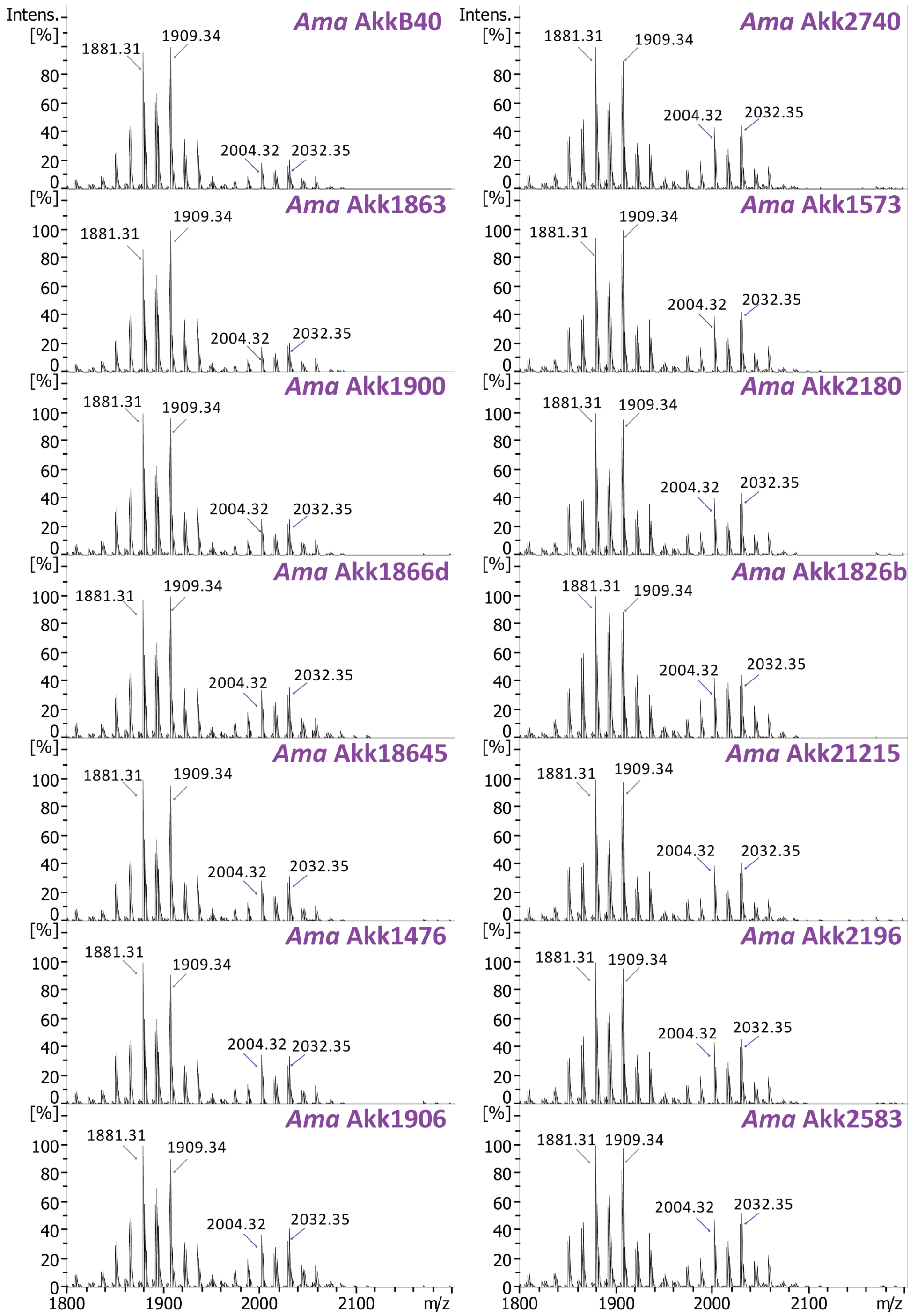

Figure S4. Lipid profiles of *Akkermansia massiliensis* (*Ama*) isolates grown in porcine gastric mucin medium to early stationary phase. Mass spectra obtained following FLAT in the negative ion mode on the timsTOF *flex* MALDI-2 (Bruker) from stationary phase cultures. Each mass spectrum is representative of three biological replicates; measured intensity is represented in relative units (%). Strain description can be found in **Table 1**.

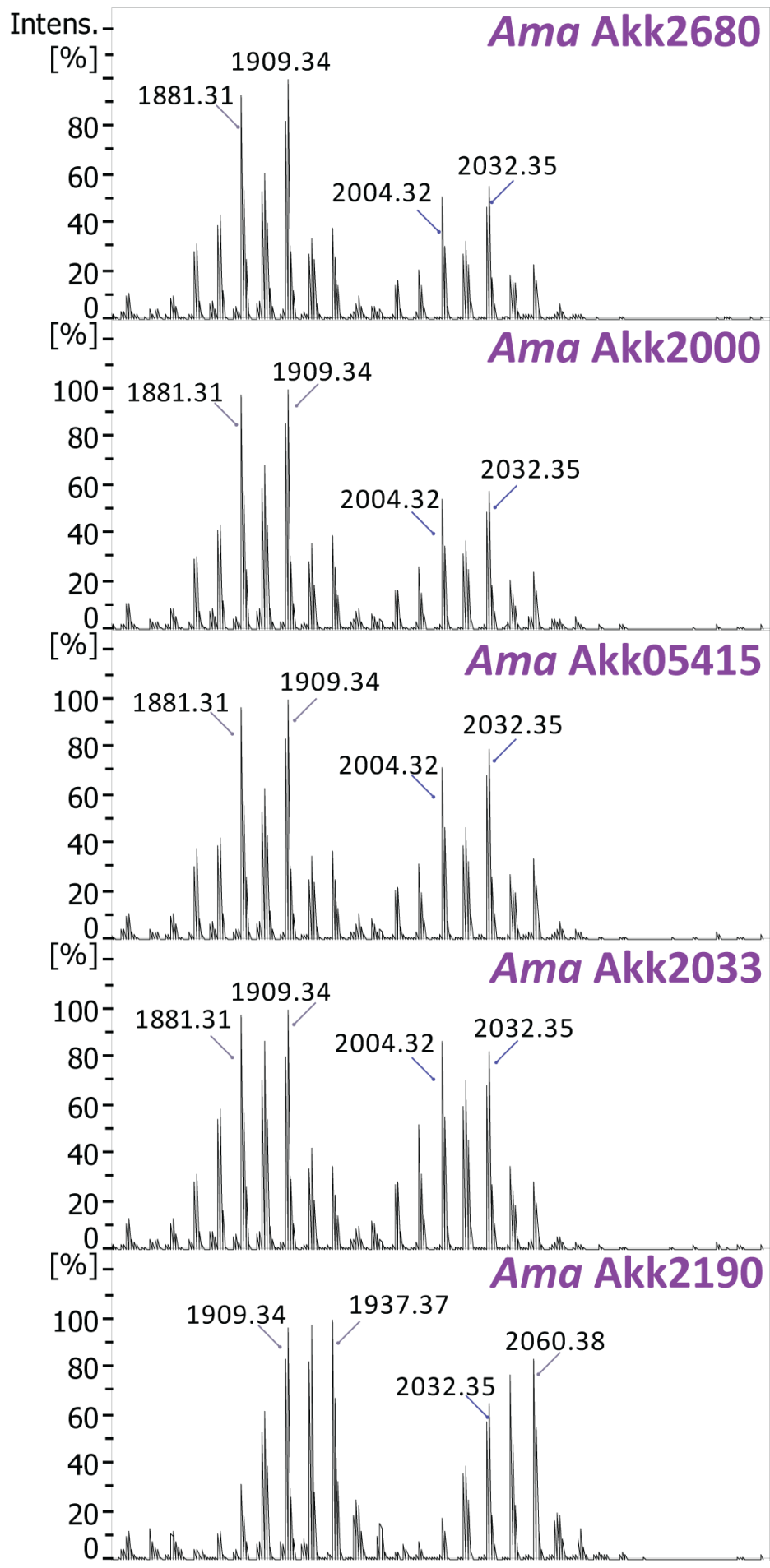

Figure S4. Lipid profiles of *Akkermansia massiliensis* (*Ama*) isolates grown in porcine gastric mucin medium to early stationary phase (continued).

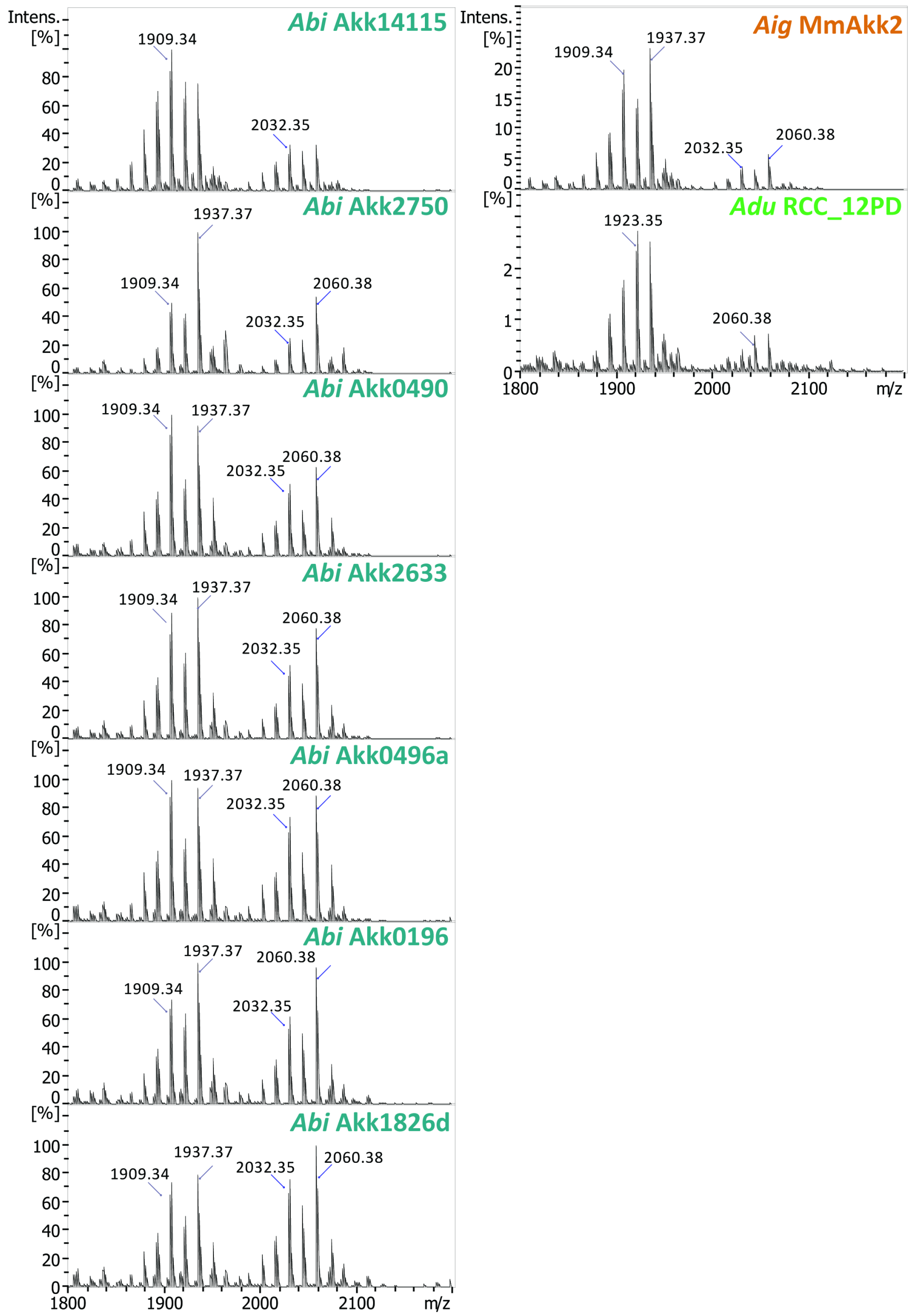

Figure S5. Lipid profiles of *Akkermansia biwaensis* (*Abi*), *Akkermansia ignis* (*Aig*) and *Akkermansia durhami* (*Adu*) isolates grown in porcine gastric mucin medium to early stationary phase. Mass spectra obtained following FLAT in the negative ion mode on the timsTOF *flex* MALDI-2 (Bruker) from stationary phase cultures. Each mass spectrum is representative of three biological replicates; measured intensity is represented in relative units (%). Strain description can be found in **Table 1**.

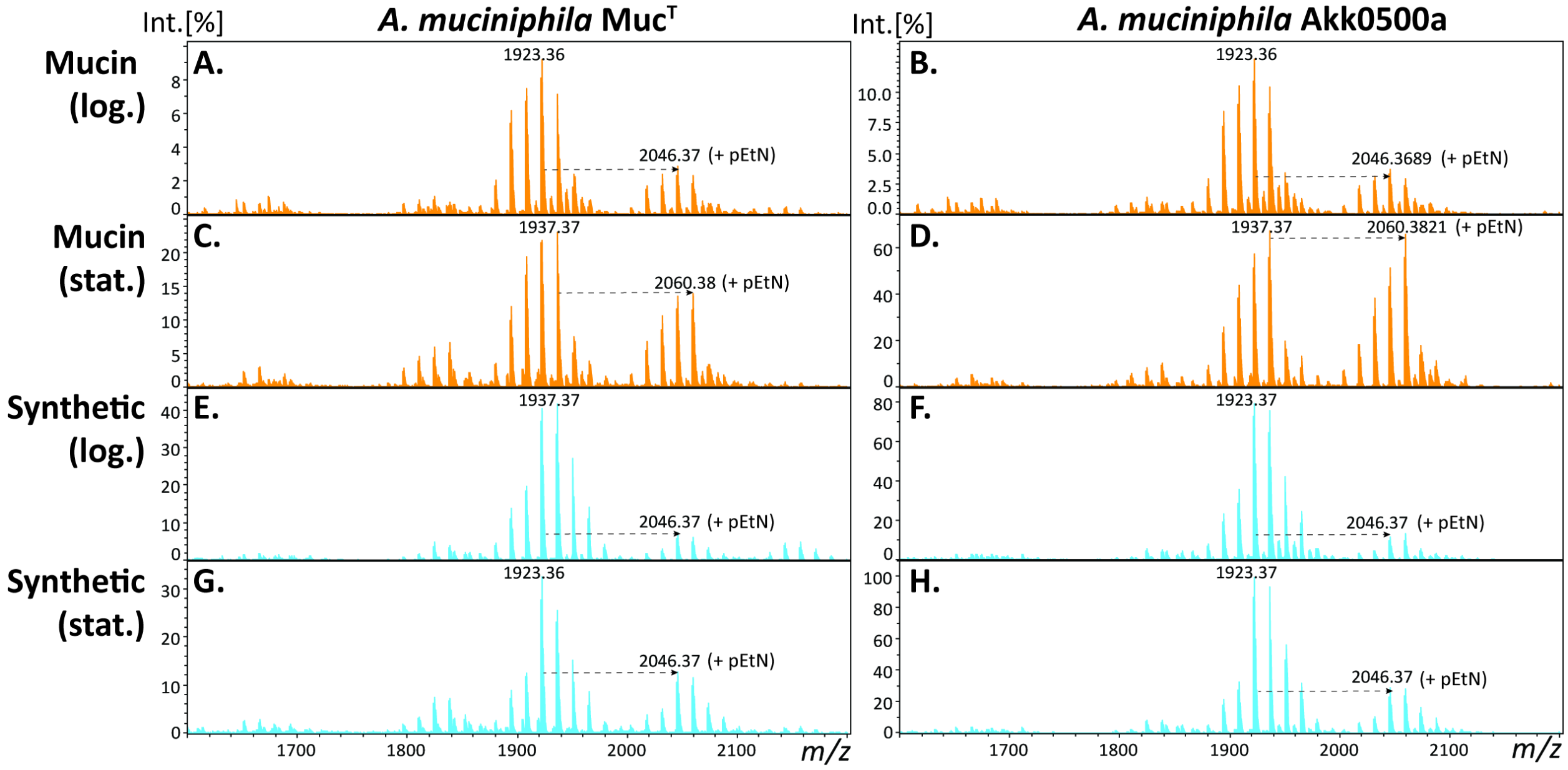

Figure S6. Lipid A profiles of representative *Akkermansia* isolates in different growth conditions. A-D. Mucin medium. **E-H.** Synthetic medium. **A.**, **C.**, **E.**, and **G.** *A. muciniphila* ssp. *muciniphila* Muc^{T} **D. B.**, **D.**, **F.**, and **H.** *A. muciniphila* ssp. *muciniphila* Akk0500a. Samples were collected at early logarithmic phase (A-B. and E-F.) and early stationary phase (C-D. and G-H.). Data were collected in the negative ion mode on a timsTOF *flex* MALDI-2 instrument. Representative spectra from three biological replicates are shown.

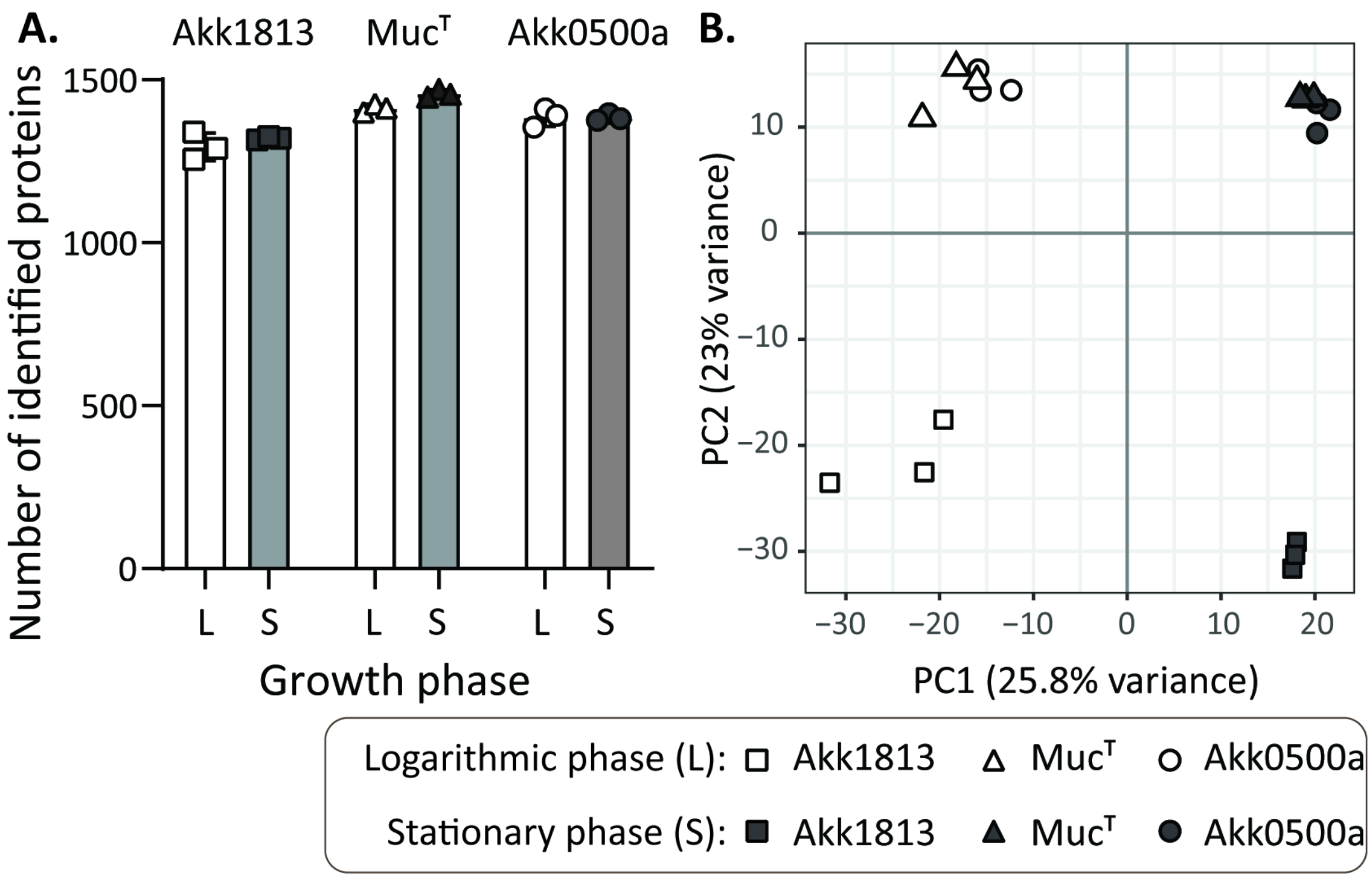

Figure S7. Overview of the proteomics data set. A. Total number of identified proteins in each sample (N=3 biological replicates per strain). **B. Principal component analysis (PCA).** Shapes stand for individual biological replicates. All detected proteins were included in the analysis. PC – principal component; L – early logarithmic (shapes with white fill); S – early stationary (shapes with color fill).

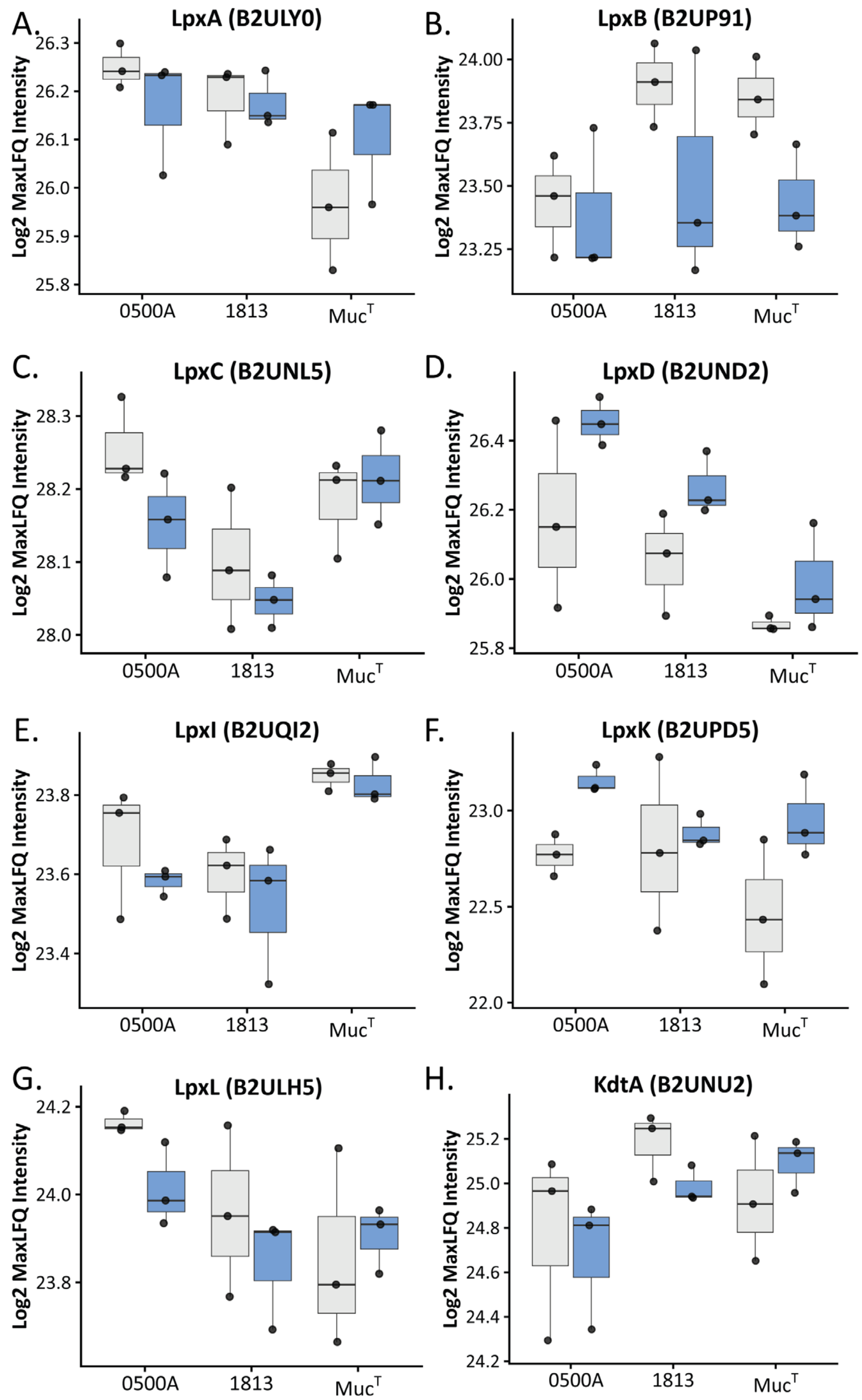

Figure S8. Relative abundance of proteins involved in lipid A biosynthesis (Raetz pathway) in representative *Akkermansia muciniphila* isolates. A-H. Log2 MaxLFQ intensities for individual enzymes of the Raetz pathway: **A.** LpxA (B2ULY0), **B.** LpxB (B2UP91), **C.** LpxC (B2UNL5), **D.** LpxD (B2UND2), **E.** LpxI (B2UQ12), **F.** LpxK (B2UPD5), **G.** LpxL (B2ULH5), and **H.** KdtA (B2UNU2). Light gray boxes represent the early logarithmic phase, and blue boxes represent the early stationary phase for each indicated isolate.

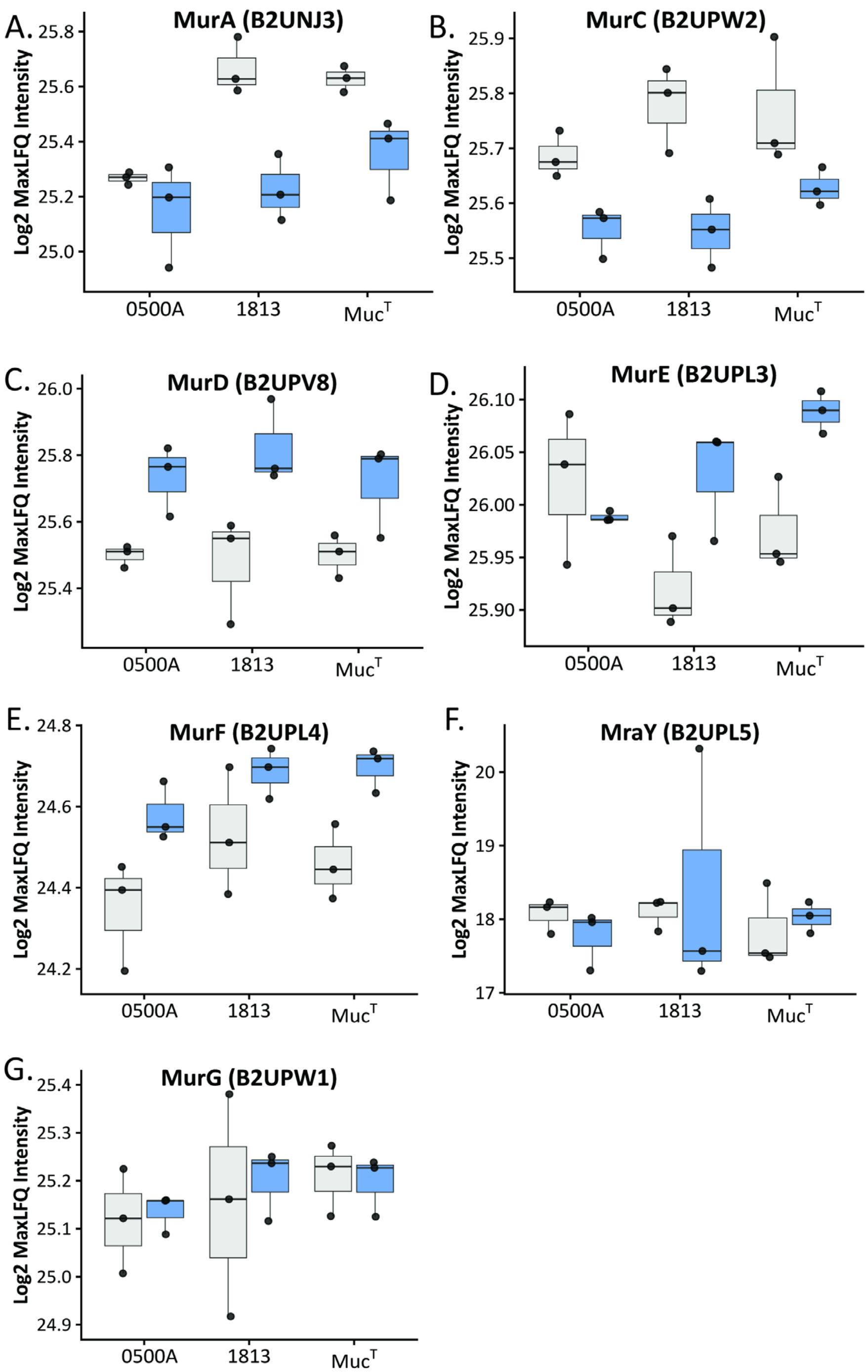

Figure S9. Relative abundance of proteins involved in peptidoglycan biosynthesis in representative *Akkermansia muciniphila* isolates. A-G. Log2 MaxLFQ intensities for individual enzymes involved in peptidoglycan synthesis: **A.** MurA (B2UNJ3), **B.** MurC (B2UPW2), **C.** MurD (B2UPV8), **D.** MurE (B2UPL3), **E.** MurF (B2UPL4), **F.** MraY (B2UPL5), and **G.** MurG (B2UPW1). Light gray boxes represent the early logarithmic phase, and blue boxes represent the early stationary phase for each indicated isolate.
